# A Novel Theranostic Strategy for Malignant Pulmonary Nodules by Targeted CECAM6 with ^89^Zr/^131^I‐Labeled Tinurilimab

**DOI:** 10.1002/advs.202415689

**Published:** 2025-04-03

**Authors:** Chongyang Chen, Keying Zhu, Jing Wang, Donghui Pan, Xinyu Wang, Yuping Xu, Junjie Yan, Lizhen Wang, Min Yang

**Affiliations:** ^1^ NHC Key Laboratory of Nuclear Medicine Jiangsu Key Laboratory of Molecular Nuclear Medicine Jiangsu Institute of Nuclear Medicine Wuxi Jiangsu 214063 China; ^2^ School of Pharmacy Nanjing Medical University Nanjing 211166 China; ^3^ Wuxi Xishan NJU Institute of Applied Biotechnology Anzhen Street, Xishan District Wuxi 214101 China

**Keywords:** ^89^Zr/^131^I‐labeled tinurilimab, CEACAM6, LUAD, malignant pulmonary nodules, PET/MR, theranostics

## Abstract

Lung adenocarcinoma (LUAD) constitutes a major cause of cancer‐related fatalities worldwide. Early identification of malignant pulmonary nodules constitutes the most effective approach to reducing the mortality of LUAD. Despite the wide application of low‐dose computed tomography (LDCT) in the early screening of LUAD, the identification of malignant pulmonary nodules by it remains a challenge. In this study, CEACAM6 (also called CD66c) as a potential biomarker is investigated for differentiating malignant lung nodules. Then, the CEACAM6‐targeting monoclonal antibody (mAb, tinurilimab) is radiolabeled with ^89^Zr and ^131^I for theranostic applications. In terms of diagnosis, machine learning confirms CEACAM6 as a specific extracellular marker for discrimination between LUAD and benign nodules. The ^89^Zr‐labeled mAb is highly specific uptake in CEACAM6‐positive LUAD via positron emission tomography (PET) imaging, and its ability to distinguish in malignant pulmonary nodules are significantly higher than that of ^18^F Fluorodeoxyglucose (FDG) by positron emission tomography/magnetic resonance (PET/MR) imaging. While the ^131^I‐labeled mAb serving as the therapeutic aspect has significantly suppressed tumor growth after a single treatment. These results proves that ^89^Zr/^131^I‐labeled tinurilimab facilitates the differential capacity of malignant pulmonary nodules and radioimmunotherapy of LUAD in preclinical models. Further clinical evaluation and translation of this CEACAM6‐targeted theranostics may be significant help in diagnosis and treatment of LUAD.

## Graphical abstract

This study investigates the integrated diagnostic and therapeutic strategy utilizing ^89^Zr/^131^I‐labeled tinurilimab for the management of malignant lung nodules, with a particular focus on lung adenocarcinoma (LUAD). CEACAM6, which is highly expressed in most LUAD patients, activates the Src/FAK signaling pathway, thereby promoting cell proliferation and metastasis. The use of ^89^Zr‐Df‐tinurilimab enables the early identification of malignant nodules during LUAD progression, facilitating timely differentiation of malignant lesions. Following this, ^131^I‐tinurilimab delivers targeted radioimmunotherapy to inhibit tumor cell growth, aiming to achieve early diagnosis and intervention in LUAD. This approach holds promise as a potential method for enhancing the early detection and treatment of LUAD.

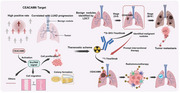

## 1 Introduction

Lung cancer remains the predominant cause of cancer incidence and mortality worldwide.^[^ ^1^ ^]^ With a 5‐year survival rate of less than 20%,^[^ ^2^ ^]^ lung adenocarcinoma (LUAD) is the most prevalent type of lung cancer, accounting for ≈ half of all lung cancers.^[^ ^3^ ^]^ Compared with other histological types of lung cancer, the proportion of LUAD among non‐smokers is significantly higher, and its pathogenesis is complex, being one of the major diseases that urgently require research.^[^ ^4^ ^]^ Despite the diversity of LUAD treatment options, such as surgery, radiotherapy, chemotherapy, targeting, and immunotherapy, the overall prognosis for LUAD patients is still poor,^[^ ^5^ ^]^ mainly because ≈75% of LUAD patients are diagnosed at an advanced stage of the disease (stage III–IV), where the 5‐year survival rate for stage IV is less than 10%. When the disease is at stage I, the 5‐year survival rate improves significantly, ranging from 68 to 92%.^[^ ^6^ ^]^ Thus, the early detection of LUAD represents the most efficacious measure for reducing LUAD mortality. The precise identification of malignant lung nodules constitutes the sole approach to detect LUAD at an early stage.

Currently, low‐dose computed tomography (LDCT) has been prevalently employed for the early screening of LUAD, and can efficaciously diminish the mortality among high‐risk groups.^[^ ^7^ ^]^ Nevertheless, LDCT remains incapable of differentiating benign from malignant lung nodules in 50–76% of screened lung nodules^[^ ^8^ ^]^ and ≈18% of patients diagnosed through LDCT will be overdiagnosed during follow‐up surveillance.^[^ ^9^ ^]^ These researches indicated that although LDCT can timely and conveniently detect early pulmonary nodules, there are still deficiencies in differentiating benign and malignant nodules. In contrast, ^18^F‐Fluorodeoxyglucose (FDG) positron emission tomography/computed tomography (PET/CT), the best non‐invasive examination method, has been recommended by clinical guidelines to identify benign and malignant pulmonary nodules, and the differential efficacy of malignant nodules exceeding 8 mm was significantly higher than that of enhanced CT.^[^ ^10^ ^]^ However, due to its low specificity, it fails to accurately identify some pulmonary nodules with infection, inflammation, or low glucose metabolism.^[^ ^11^ ^]^ Hence, early and precise identification malignant pulmonary nodules could be the most effective way to prevent overtreatment and lower the mortality of LUAD. Exploring the specific biomarkers of pulmonary nodule malignant transformation at the early stage of LUAD will offer a novel and more efficient strategy for the early diagnosis and treatment of LUAD.

Recently, an increasing number of omics analyses have disclosed the biomarkers of LUAD, such as prognostic plasma biomarker HSP90β,^[^ ^12^ ^]^ MMP11 as a biomarker for early detection,^[^ ^4^ ^]^ etc. However, these findings were based on LUAD tissue and adjacent tissue, and absence of data for comparison with benign nodules. Generally, discovering specific biomarkers that differentiate lung nodules from LUAD will be of paramount significance for the diagnosis and treatment of LUAD. Previous research has reported that carcinoembryonic antigen‐related cell adhesion molecule 6 (CEACAM6) was overexpressed in LUAD compared to benign pulmonary nodules,^[^ ^13^ ^]^ while the specificity of it to distinguish benign nodules from malignant tumor (LUAD) remains to be further clarified. In clinical studies, CEACAM6 was discovered to be highly expressed in non‐small cell lung cancer and overexpressed in over 80% of LUAD patients.^[^ ^14^ ^]^ Among the entire onco‐embryonic antigen gene family, CEACAM6 is the most characteristic biomarker of numerous aggressive tumors.^[^ ^15^, ^16^ ^]^ Therefore, CEACAM6 may serve as a potential biomarker and target for theranostics of LUAD.

In therapeutic aspect, the preferred treatment for patients with malignant pulmonary nodules is surgical radical resection, which typically involves lobectomy and lymph node dissection and carries a mortality rate of ≈1 to 4%.^[^ ^17^ ^]^ For patients with physiological dysfunctions such as cardiopulmonary insufficiency, stereotactic body radiotherapy (SBRT) or ablation therapy are recommended. However, these treatments either involve invasiveness with an associated mortality risk or may be less effective. CEACAM6 has been reported to be an immune checkpoint modulator capable of inhibiting the antitumor activity of effector T cells.^[^ ^18^ ^]^ The CAR‐T therapy targeting CEACAM6 potently inhibits the growth of pancreatic cancer tumor models both in vitro and in vivo.^[^ ^19^ ^]^ Tinurilimab, an anti‐CEACAM6 monoclonal antibody has embarked on phase I clinical studies for the treatment of solid tumors.^[^ ^20^ ^]^ Owing to the heterogeneity of the tumor or the complexity of the tumor microenvironment, the therapeutic effect of immunotherapy alone is frequently restricted. Meanwhile, radioimmunotherapy, as a novel tumor treatment integrating nuclear medicine and immunology, is capable of directly regulating T lymphocytes to eliminate the tumor and enhance the efficacy in combination with immunosuppressive agents.^[^ ^21^, ^22^ ^]^ For instance, the radioimmunotherapy targeting CD38 suppresses tumor growth of lymphoma,^[^ ^23^ ^] 131^I‐tositumomab therapy for follicular B‐cell lymphoma.^[^ ^24^ ^]^ In addition, immuno‐positron emission tomography (immunoPET) integrates the high sensitivity of PET imaging with the specificity of monoclonal antibodies (mAbs), providing an ideal approach for non‐invasive assessment of target expression.^[^ ^25^ ^]^ Therefore, immunoPET and radioimmunotherapy targeting CEACAM6 could potentially prove beneficial for the differential diagnosis of malignant pulmonary nodules and the treatment of LUAD.

Here, machine learning was employed to demonstrate the capability of CEACAM6 in distinguishing benign nodules from LUAD. The ^89^Zr/^131^I‐labled tinurilimab was comprehensively investigating the imaging of identification of malignant pulmonary nodules, the treatment and toxicity in LUAD mouse model. Through this study, we expect to establish a promising approach for the precise theranostics of malignant pulmonary nodules at the early stage of LUAD in future clinical practice.

## 2 Results

### 2.1 Machine Learning Confirmed CEACAM6 as a Specific Cell Surface Biomarker for Distinguishing Benign Lung Nodules from LUAD

**Figure 1:**
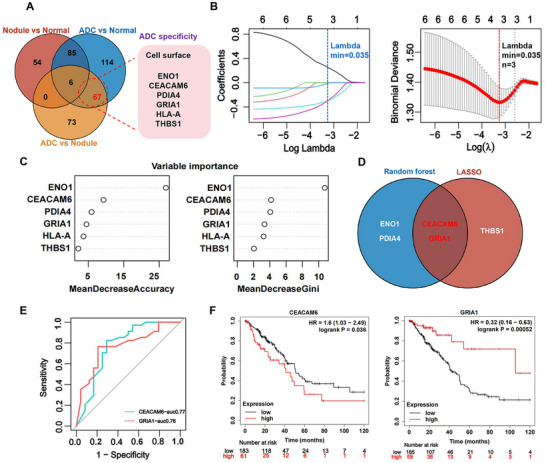
CEACAM6 was suitable to serve as a specific biomarker for discriminating benign lung nodules from LUAD. A) Venny analysis the differentially expressed proteins between different compared groups (Nodule vs Normal, ADC vs Normal, and ADC vs Nodule), and the ADC‐specific proteins enriched in cell surface were shown in the right. B–D) Two cell surface proteins were identified as diagnostic biomarkers by LASSO regression and random forest method. The optimum penalty parameter λ was labeled and the hub biomarkers were shown in Venny graph.) ROC curves were evaluated to assess the diagnostic efficacy of two proteins: CEACAM6 (AUC = 0.77), GRIA1 (AUC = 0.76). F) Survival analysis of LUAD patients characterized by low tumor mutation load and disparate mRNA expression levels of CEACAM6 or GRIA1 via pan‐cancer RNA‐seq datasets from Kaplan‐Meier plotter.

Despite the fact that a multitude of studies have disclosed molecules that were differentially expressed in LUAD, scant few have contrasted proteins that were differentially expressed in benign nodules and LUAD, or employed machine learning (ML) to filter out LUAD‐specific biomarkers for the identification of malignant pulmonary nodules. Here a proteomic dataset of benign lung nodules and LUAD derived from previous study^[^ ^13^ ^]^ was analyzed by ML to identify biomarkers (especially cell surface proteins) that may distinguish benign nodules from LUAD. After differential expression (DE) analysis, the changed proteins form compared groups of benign nodules, adenocarcinoma and normal (nodule vs normal, adenocarcinoma (ADC) vs normal, ADC vs nodule) were analyzed to filter out ADC specific proteins. To identify appropriate targets for PET tracer development, the ADC specific proteins were enrichment analysis of cellular components to obtain proteins expressed on the cell surface. A total 67 DE proteins were specifically over expressed in ADC, among which six proteins were expressed on the cell surface (**Figure** **1**A). Then, the ML including least absolute shrinkage and selection operator (LASSO) and random forest (RF) were used to identify specific biomarkers from among these six extracellular proteins for discriminating benign nodules from LUAD. The LASSO analysis filtered out three potential biomarkers from six extracellular proteins (Figure 1B), whereas RF analysis identified four important biomarkers from the same set (Figure 1C). The Venny analysis demonstrated that two proteins (CEACAM6 and GRIA1) screened out form LASSO and RF approaches (Figure 1D) could be the crucial biomarkers. Additionally, the ROC (receiver operating characteristic) curve demonstrated that CEACAM6 and GRIA1 possessed an excellent capability to distinguish benign nodules from LUAD, with the AUC being 0.77 and 0.76, respectively (Figure 1E).

To investigate the significance of CEACAM6 and GRIA1 in the prognosis of LUAD, the mRNA data form Kaplan‐Meier plotter were employed to analyze the overall survival between these two proteins in LUAD with a low tumor mutation burden (TMB). The rationale for enrolling patients with a low TMB lies in the fact that the efficacy of immunotherapy is often compromised when the TMB is relative lower.^[^ ^26^ ^]^ Thus, identifying molecules significantly correlated with prognosis in patients with low TMB can facilitate the development of novel targets or strategies for immunotherapy. After overall survival (OS) analysis, the overexpression of CEACAM6 was significantly correlated with the decreased OS, while the overexpression of GRIA1 was significantly correlated with the increased OS (Figure 1F). Therefore, CEACAM6 is capable of reflecting the advancement of tumor more precisely and is appropriate to be utilized as a biomarker for the diagnosis or prognosis of LUAD as well as a cell surface PET tracer target.

Furthermore, to elucidate the relevance of CEACAM6 in LUAD, we investigated its impact on the Src/FAK signaling pathway, which has been reported to regulate the proliferation and migration of primary LUAD cells. As shown in Figure S1A (Supporting Information), CEACAM6 knockdown significantly inhibited the activation of the Src/FAK signaling pathway in A549 cells, as evidenced by a significantly reduction in p–Src and p–FAK levels. Moreover, CEACAM6 knockdown also led to a significant suppression of A549 cell migration (Figure S1B, Supporting Information). All these results indicated that CEACAM6 plays a crucial role in the development and progression of LUAD.

### 2.2 ^89^Zr Radiolabeling Showed No Influence on the Targeting Specificity and Affinity of Tinurilimab In Vitro

**Figure 2:**
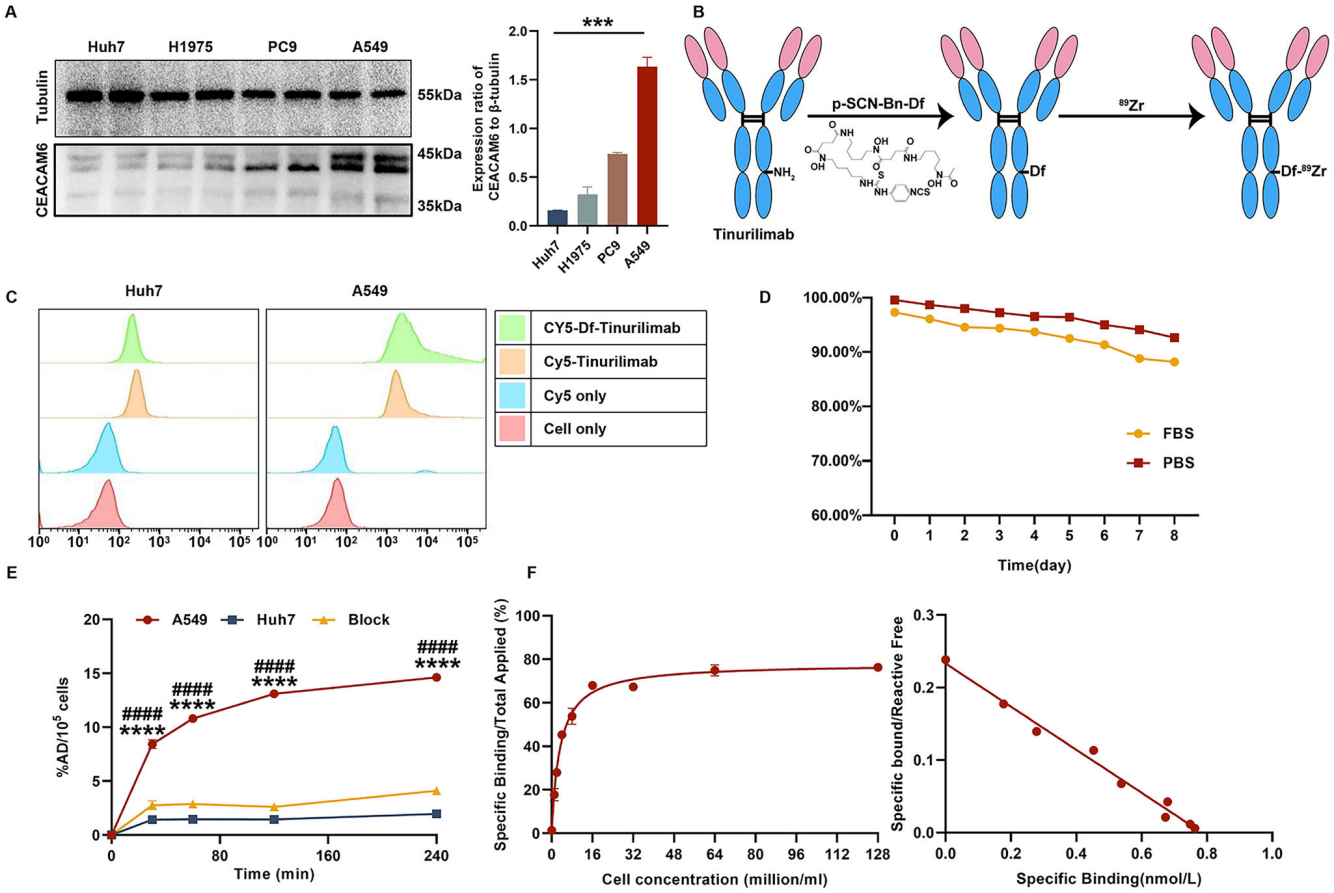
Scheme for the study of ^89^Zr radiolabeled tinurilimab and in vitro evaluation. A) Western blots and quantify of CEACAM6 expression in LUAD tumor cell (H1975, PC9, and A549) and hepatocellular carcinoma cell (Huh7). B) The general procedure of 89Zr‐labed tinurilimab. C) Flow cytometry analysis the cellular binding affinity of Cy5‐labeled Df‐conjugated tinurilimab. D) The in vitro stability analysis of ^89^Zr‐Df‐tinurilimab in PBS and plasma within 1 to 8 days post‐incubation. E) Uptake of ^89^Zr‐Df‐tinurilimab in A549 and Huh7 tumor cell. F) Lindmo assay the immunogenicity of ^89^Zr‐Df‐tinurilimab in A549 cell and the scatchard plots of ^89^Zr‐Df‐tinurilimab binding to A549 cell. All data are expressed as mean ± SEM. ^***^ *p* < 0.001, ^****^ *p* < 0.0001, A549 vs Huh7. ^####^ *p* < 0.0001, A549 vs Block. *n* = 4 for each group.

To ascertain the pivotal role of CEACAM6 in the identification and advancement of malignant pulmonary nodules, ^89^Zr‐radiolabeled CEACAM6‐targeted antibody (^89^Zr‐Df‐tinurilimab) was utilized to undertake non‐invasive identification and progression monitoring of malignant pulmonary nodules. Western blotting and immunofluorescence analysis revealed that CEACAM6 was significantly overexpressed in A549 cells compared with other tumor cells (such as Huh7, PC9, and H1975), and the Huh7 cell line showed an extremely limited expression of this protein. (**Figure** **2**A; Figure S2A, Supporting Information). Thus, A549 and Huh7 were respectively employed in subsequent CEACAM6‐positive and CEACAM6‐negative tumor cells. The development of ^89^Zr‐Df‐tinurilimab as an immunoPET agent was accomplished through covalent conjugation of the chelator Df on non‐site‐specific lysine residues followed by radiolabeling with ^89^Zr (Figure 2B). Flow cytometry analysis revealed that the Df modification exerted no influence on the antibody affinity of tinurilimab (Figure 2C). After labeling with ^89^Zr, the ^89^Zr‐Df‐tinurilimab showed a radiochemical purity greater than 95%, and specific activities of 111 MBq mg^−1^ (Figure S2B, Supporting Information). And ^89^Zr‐Df‐Tinurilimab exhibited an excellent in vitro stability, displaying > 95% in PBS and plasma over 8 days (Figure 2D). Additionally, the cell uptake analysis demonstrated that ^89^Zr‐Df‐tinurilimab was specifically uptake in A549 cells compared with Huh7 cells (Figure 2E). The uptake of ^89^Zr‐Df‐tinurilimab in A549 cells at 30, 60, 120, and 240 min was 8.45 ± 0.41, 10.82 ± 0.24, 13.10 ± 0.38, and 14.62 ± 0.25% AD/10^5^ cells, respectively, which was significantly higher than that in Huh7 cells (1.42 ± 0.19, 1.45 ± 0.09, 1.43 ± 0.09, and 1.96 ± 0.10% AD/10^5^ cells, respectively). However, the uptake of ^89^Zr‐Df‐tinurilimab was significantly blocked by the presence of excess unlabeled tinurilimab, confirming ^89^Zr‐Df‐tinurilimab binding specificity to the antigen of CEACAM6. The binding characteristics of ^89^Zr‐Df‐tinurilimab to CEACAM6 were investigated using a Lindmo cell binding assay and scatchard analysis (Figure 2F). Notably, ^89^Zr‐Df‐tinurilimab exhibited high affinity binding to CEACAM6, with binding affinity of 0.30 nm and immunoreactivity of 76.29 ± 0.13%. All the in vitro experiments demonstrated that ^89^Zr labeling did not modify the specificity and affinity of tinurilimab in targeting CEACAM6.

### 2.3 ^89^Zr‐Df‐Tinurilimab Specifically Targeted CEAMCA6‐Positive Xenografts In Vivo

**Figure 3:**
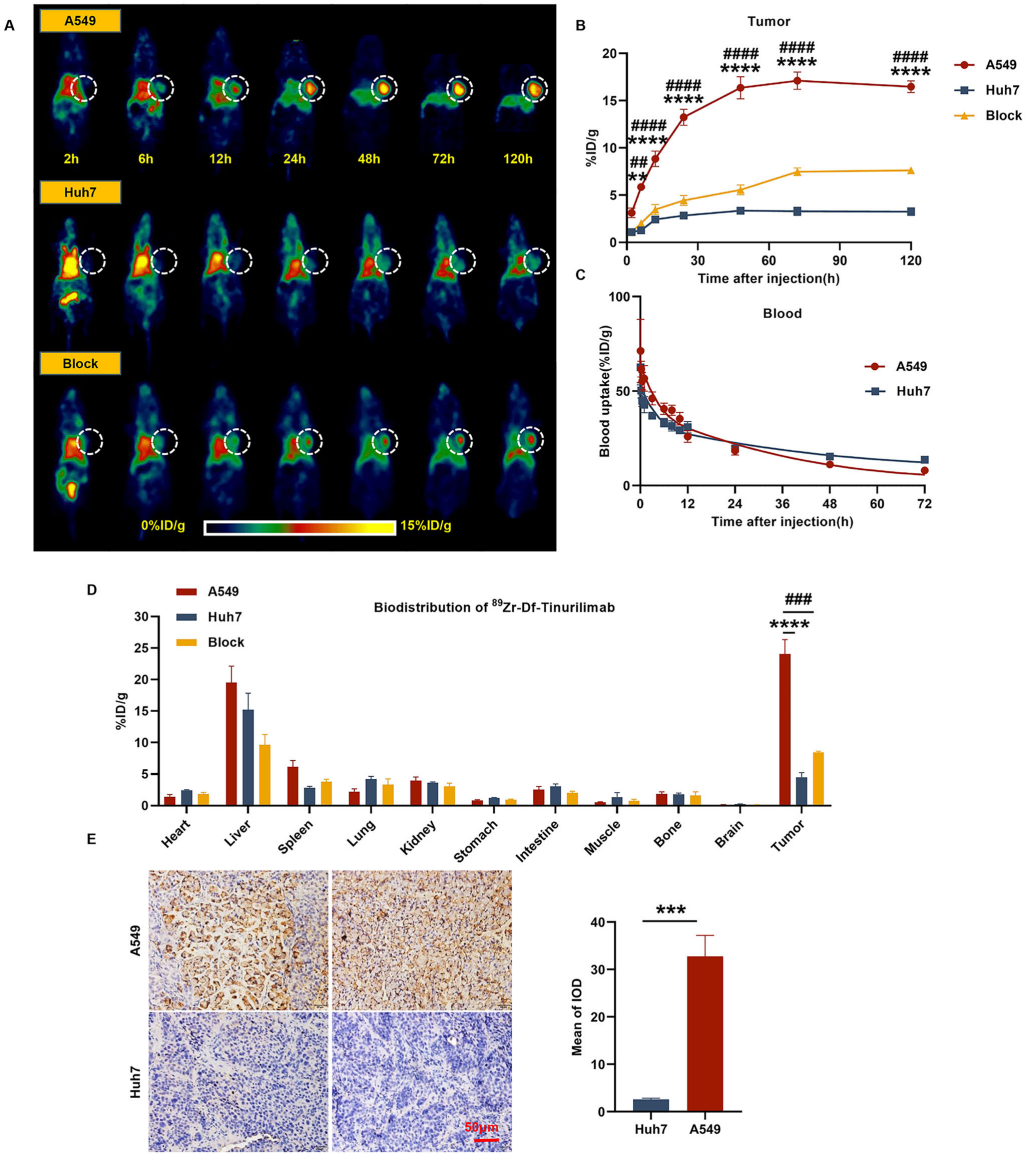
^89^Zr‐Df‐tinurilimab exhibited specific uptake in CEACAM6‐positive tumor‐bearing mice. A) The maximum intensity projection (MIP) PET images depicting the uptake of ^89^Zr‐Df‐tinurilimab in CECAMA6‐positive tumor (A549), CECAMA6‐negative tumor (Huh7), and the unlabeled Df‐tinurilimab group (Block). B) Quantitative analysis the ROI (region of interest) obtained from PET imaging in groups of A549, Huh7, and Block. C) The blood pharmacokinetics of ^89^Zr‐Df‐tinurilimab in A549 and Huh7 tumor models. D) Ex vivo biodistributions of ^89^Zr‐Df‐tinurilimab in these experiment groups at 120 h post‐injection. E) Immunohistochemical analysis of CEACAM6 expression in tumor tissues of A549 and Huh7. All data are expressed as mean ± SEM. ^**^ *p* < 0.01, ^***^ *p* < 0.001, ^****^ *p* < 0.0001, A549 vs Huh7. ^##^ *p* < 0.01, ^###^ *p* < 0.001, ^####^ *p* < 0.0001, A549 vs Block. *n* = 4 for each group.

Excellent in vivo imaging performance is a crucial indicator for achieving non‐invasive monitoring of CEACAM6. The xenograft tumor models of CEACAM6‐positive (A549) and CEACAM6‐negative (Huh7) were established to further assess the specificity of ^89^Zr‐Df‐tinurilimab in targeting CEACAM6‐positive tumors in vivo. Following microPET analysis, the uptake of ^89^Zr‐Df‐tinurilimab in A549 xenografts was significantly increased from 6 h post‐injection (PI) in comparison to the Huh7 xenografts (**Figure** **3**A). The ROI analysis showed that the tumor uptake increased from 3.12 ± 0.50 to 16.48 ± 0.62% ID g^−1^ in A549 xenografts, whereas there was only a slight increase from 1.09 ± 0.29 to 3.25 ± 0.40% ID g^−1^ in Huh7 xenografts from 2 to 120 h (Figure 3B). Nevertheless, after co‐injection of unlabeled tinurilimab (Df‐tinurilimab), the uptake of ^89^Zr‐Df‐tinurilimab was significantly decreased in A549 xenografts compared with unblock groups. In vivo pharmacokinetic analysis revealed that ^89^Zr‐Df‐tinurilimab had a distribution half‐life of 0.19 h and a clearance half‐life of ≈17.25 h, suggesting that its slower metabolic characteristics might be conducive to subsequent radioimmunotherapy (Figure 3C).

In addition, the ex vivo biodistribution analysis reconfirmed that ^89^Zr‐Df‐tinurilimab exhibited specific uptake in A549 xenografts when compared with Huh7 xenografts and the Df‐tinurilimab blocked groups (Figure 3D). At 120 h post‐injection, the uptake of ^89^Zr‐Df‐tinurilimab in A549 xenografts was 24.09 ± 2.26% ID g^−1^, which was significantly higher than that in Huh7 xenografts (4.52 ± 0.72% ID g^−1^) and Df‐tinurilimab blocked groups (8.46 ± 0.17% ID g^−1^). Subsequent to the euthanasia of mice, immunohistochemical analysis demonstrated that the expression of CEACAM6 in A549 xenografts was indeed significantly higher than that in Huh7 xenografts (Figure 3E). All these results indicated that ^89^Zr‐Df‐tinurilimab was specifically targeted CEAMCA6‐positive xenografts in vivo and suitable for non‐invasive real‐time monitoring of the expression of CEACAM6 in malignant nodules or LUAD.

Given the limited diversity of cell lines and animal models, the scope of the study was expanded to include two additional LUAD subtypes, PC9 and Calu‐3. The PET imaging (Figure S3A, Supporting Information) and ex vivo biodistribution (Figure S3C, Supporting Information) of ^89^Zr‐Df‐tinurilimab in these tumors were evaluated. ROI quantification analysis (Figure S3B, Supporting Information) revealed a significant accumulation of ^89^Zr‐Df‐tinurilimab in tumor regions, with enhanced uptake in comparison to surrounding tissues. Immunohistochemical staining (Figure S3D, Supporting Information) confirmed the specific localization of the tracer to tumor tissues, supporting its potential as a precise imaging tool for LUAD. The results demonstrated that ^89^Zr‐Df‐tinurilimab specifically targeted and enriched in these distinct LUAD models.

Hematoxylin and Eosin (HE) staining was performed to evaluate morphological changes in major organs. No necrosis or deformation was observed in the heart, liver, spleen, lung, or kidney, indicating that there was no significant toxicity in these tissues after the injection of ^89^Zr‐Df‐tinurilimab (Figure S4, Supporting Information).

### 2.4 ^89^Zr‐Df‐Tinurilimab Showed a More Excellent Performance in the Identification and Progression of Malignant Pulmonary Nodules

**Figure 4:**
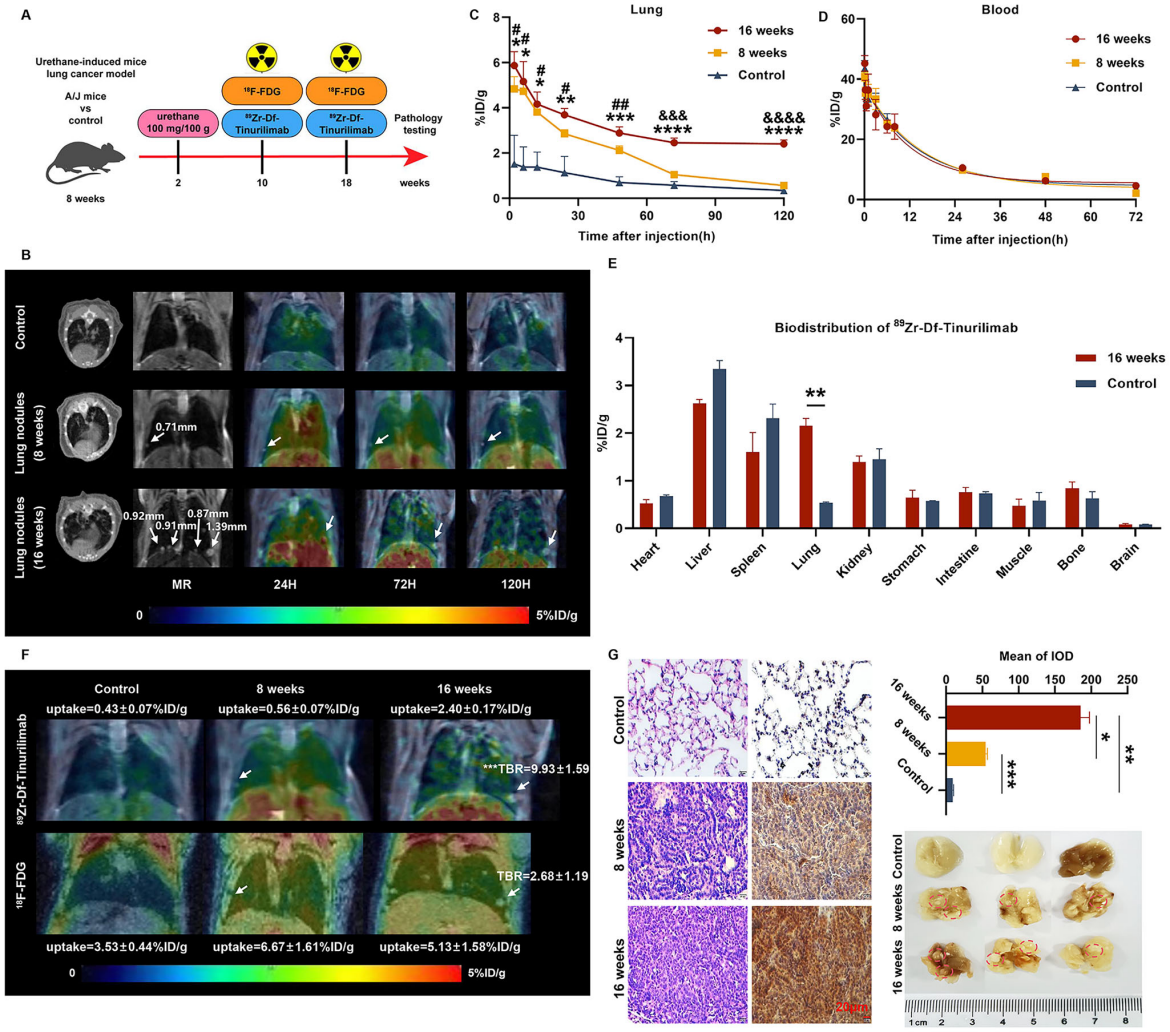
^89^Zr‐Df‐tinurilimab showed a favorable performance in minatory and identification of malignant pulmonary nodules. A) Scheme for the minatory and identification of malignant pulmonary nodules in the early stage of urethane‐induced LUAD. B) The maximum intensity projection (MIP) PET/MR images depicting the uptake of ^89^Zr‐Df‐tinurilimab in urethane treated mice at 24, 72, and 120 h post‐injection. C) After 8 weeks or 16 weeks of urethane treatment, PET imaging was performed on mice, and quantitative analysis was conducted on the ROI, then compared with the control group that was not treated. D) The blood pharmacokinetics of ^89^Zr‐Df‐tinurilimab in urethane treated or untreated mice. E) Ex vivo biodistributions of ^89^Zr‐Df‐tinurilimab in urethane treated or untreated mice at 120 h post‐injection. F) Representative PET/MR images comparing 50 min post‐injection of ^18^F‐FDG and 72 h post‐injection of ^89^Zr‐Df‐tinurilimab in lung nodules of urethane‐induced mice or uninduced mice. G) Representative images of HE, immunohistochemistry of CEACAM6 and pulmonary nodules in urethane‐treated mice. All data are expressed as mean ± SEM. ^*^ *p* < 0.05, ^**^ *p* < 0.01, ^***^ *p* < 0.001, ^****^ *p* < 0.0001, 16 weeks vs Control. ^#^ *p* < 0.05, ^##^ *p* < 0.01, 8 weeks vs Control. ^&&&^ *p* < 0.001, ^&&&&^ *p* < 0.0001, 16 weeks vs 8 weeks. *n* = 4 for each group.

After obtained and confirmed ^89^Zr‐Df‐tinurilimab was suitable for non‐invasive monitoring of CEACAM6 expression in vivo, the urethane‐induced malignant pulmonary nodule model was employed to explore the correlation between CEACAM6 expression and the progression of malignant nodule. Based on previous studies, an animal model of malignant nodules in situ evolving to the pathological phenotype of LUAD was constructed. The size of malignant nodules and the expression of CEACAM6 were respectively monitored by microCT, positron emission tomography/magnetic resonance (PET/MR) and immunohistochemistry after 8 and 16 weeks of urethane treatment, and ^18^F‐FDG was employed to compare image performance with ^89^Zr‐Df‐tinurilimab (**Figure** **4**A). Compared with untreated mice, the size and quantity of pulmonary nodules in the lung were significantly increased in urethane‐treated mice through microCT and MR screening. Moreover, PET/MR imaging indicated that ^89^Zr‐Df‐tinurilimab was conspicuously accumulated in urethane‐treated mice (Figure 4B). After quantitative ROI analysis, the uptake of ^89^Zr‐Df‐tinurilimab in the lungs of 16‐weeks urethane‐treated mice at 72 and 120 h was 2.46 ± 0.20 and 2.40 ± 0.17% ID g^−1^, respectively, which was significantly higher than that in the lungs of control mice (0.57 ± 0.16 and 0.34 ± 0.07% ID g^−1^, respectively) (Figure 4C). Blood pharmacokinetic analysis revealed that the blood half‐life of ^89^Zr‐Df‐tinurilimab was consistent among different groups of mice, thereby demonstrating that the differential uptake of ^89^Zr‐Df‐tinurilimab in the lungs was attributed to the varying expression levels of CEACAM6 (Figure 4D). In addition, the ex vivo biodistribution analysis at 120 h post‐injection revalidated that ^89^Zr‐Df‐tinurilimab was significantly taken up in the lungs of 16‐weeks urethane‐treated mice compared with control (2.16 ± 0.15 vs 0.54 ± 0.02% ID g^−1^), and its uptake showed no disparity in other organs between any compared groups (Figure 4E). This result suggested that ^89^Zr‐Df‐tinurilimab had the capability to monitor the progression of diseases in lung nodules.

Accurate identification of benign and malignant nodules is a prerequisite for the early diagnosis of LUAD. ^18^F‐FDG PET constitutes a clinical guideline for the identification of malignant nodules in early stage of LUAD. However, the PET/MR analysis showed that the uptake target‐to‐background ratio (TBR) of ^18^F‐FDG in malignant nodules (with a diameter of 1.39 mm) of urethane treated mice was significantly lower than that of ^89^Zr‐Df‐tinurilimab (2.68 ± 1.19 vs 9.93 ± 1.59, *p* < 0.001) (Figure 4F). This result indicated that ^89^Zr‐Df‐tinurilimab exhibits a superior performance in identification and monitoring progression of pulmonary malignant nodules compared to the traditional clinical diagnostic tracer ^18^F‐FDG, and further reaffirmed the crucial role of CEACAM6 in the progression of malignant pulmonary nodules in early stage of LUAD.

To confirm the benign and malignant pulmonary nodules and the pathologic phenotype induced by urethane, the HE staining was performed. At the 8th week subsequent to urethane treatment, the lung nodules detected by microCT and MR had already turned malignant. Moreover, by the 16th week, the degree of malignancy had significantly escalated, and the nucleus had obviously enlarged, which fell into the disease phenotype of LUAD (Figure 4G). Additionally, immunohistochemical analysis showed that the expression of CEACAM6 was significantly increased in malignant pulmonary nodules of urethane treated mice compared with untreated mice. Those results indicated that the significant uptake of ^89^Zr‐Df‐tinurilimab in the lungs of urethane treated mice was due to the overexpression of CEACAM6 in malignant pulmonary nodules.

### 2.5 ^131^I Radiolabeling Preserved Specificity and Affinity of Tinurilimab

**Figure 5:**
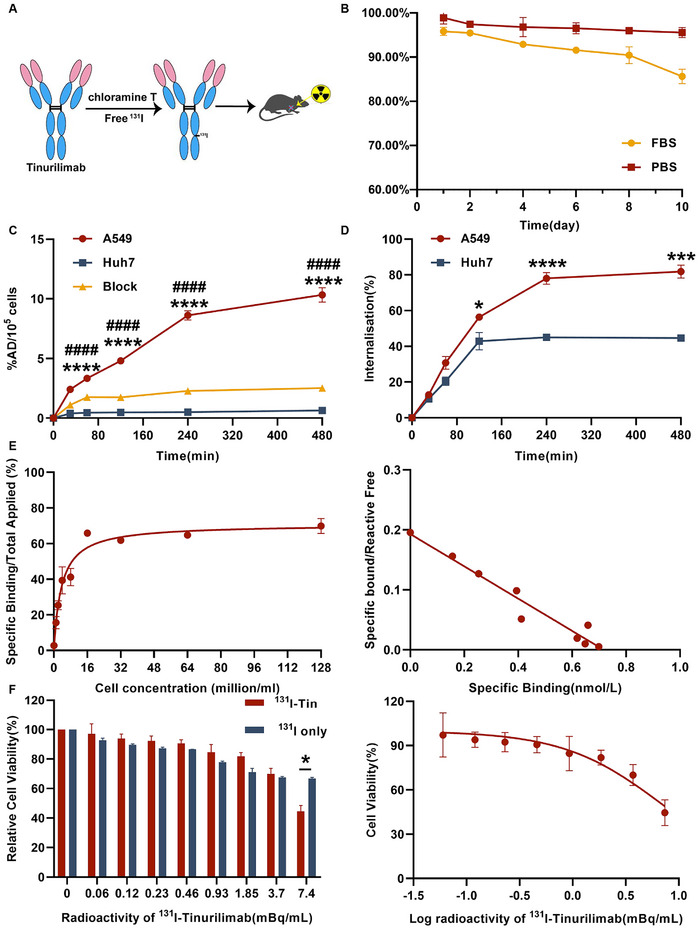
Scheme for the study of ^131^I radiolabeled tinurilimab and in vitro evaluation. A) The general procedure of ^131^I‐labed tinurilimab. B) The in vitro stability analysis of ^131^I‐tinurilimab in PBS and plasma within 1 to 10 days post‐incubation. C) Uptake of ^131^I‐tinurilimab in A549 and Huh7 tumor cell. D) Rate of cellular internalization ^131^I‐tinurilimab in A549 and Huh7 cell. E) Lindmo assay the immunogenicity of ^131^I‐tinurilimab in A549 cell and the scatchard plots of ^131^I‐tinurilimab binding to A549 cell. F) The half of the inhibition rate (IC50) of ^131^I‐tinurilimab or free ^131^I in A549 cells detected by a CCK‐8 assay. Tin: Tinurilimab. All data are expressed as mean ± SEM. ^***^ *p* < 0.001, ^****^ *p* < 0.0001, A549 vs Huh7. ^####^ *p* < 0.0001, A549 vs Block. *n* = 4 for each group.

Early diagnosis and treatment constitute the most efficacious approach for reducing the mortality of LUAD, while timely identification and treatment of malignant pulmonary nodules represent the sole approach to achieve it. After reconfirmed that CEACAM6 can be a theranostic target for pulmonary malignant nodules and the prominent and prolonged tumor uptake of ^89^Zr‐Df‐tinurilimab in the A549 tumor model, the radiotherapeutic nuclide ^131^I was used for labelling tinurilimab, followed by exploration of its therapeutic efficacy in LUAD. Radioimmunotherapy was opted for due to its capability to enhance targeted therapy, concurrently mitigate non‐specific toxic side effects and drug resistance. Furthermore, ^131^I labeling does not entail chelating agents, evading the influence on the structure of the antibody and preserving its original immunogenicity. In this case, tinurilimab was successfully labeled with ^131^I using the chloramine T method (**Figure** **5**A), with a radiochemical purity of greater than 95% (Figure S2B). And ^131^I‐tinurilimab exhibited a favorable in vitro stability (≈85%) in PBS and plasma over 10 days (Figure 5B).

The cell uptake of ^131^I‐tinurilimab in A549 cells was significantly higher than that in Huh7 cell at 480 min post‐incubation (10.33 ± 0.60% vs 0.65 ± 0.03 AD/10^5^ cells), suggesting that radiolabeling didn't affect the binding ability. After blocked with excess unlabeled tinurilimab, the uptake was significantly decreased to 2.51 ± 0.09% AD/10^5^ cells, confirming ^131^I‐tinurilimab binding specificity to CEACAM6 antigen (Figure 5C). Moreover, the internalization of ^131^I‐tinurilimab in A549 cells was significantly increased from 56.36 ± 0.97% at 2 h to a maximum of 81.85 ± 3.64% at 8 h compared with that in Huh7 cells (42.87 ± 4.83 to 44.60 ± 1.89%) (Figure 5D), indicating a favorable internalization of ^131^I‐tinurilimab in CEACAM6‐positive tumor cell. The Lindmo assay showed that the specific binding of ^131^I‐tinurilimab was reached to 69.88 ± 2.43% in A549 cells, and the corresponding dissociation constant (Kd) was calculated to be ≈0.30 nm (Figure 5E), reproving that ^131^I labeling had no effect on the immunoreactivity of tinurilimab. Further in vitro killing experimentation showed that ^131^I‐tinurilimab was significantly reduced viability of A549 cells, with a half of inhibit rate (IC50) was 7.04 MBq mL^−1^ (Figure 5F). All these results suggested that ^131^I radiolabeling preserved specificity, affinity and immunoreactivity of tinurilimab, and ^131^I‐tinurilimab may be applicable for the therapy of CEACAM6‐positve tumor (LUAD) or malignant pulmonary nodules.

### 2.6 ^131^I‐Tinurilimab Gradually and Significantly Accumulated in CEAMCA6‐Positive Xenografts

**Figure 6:**
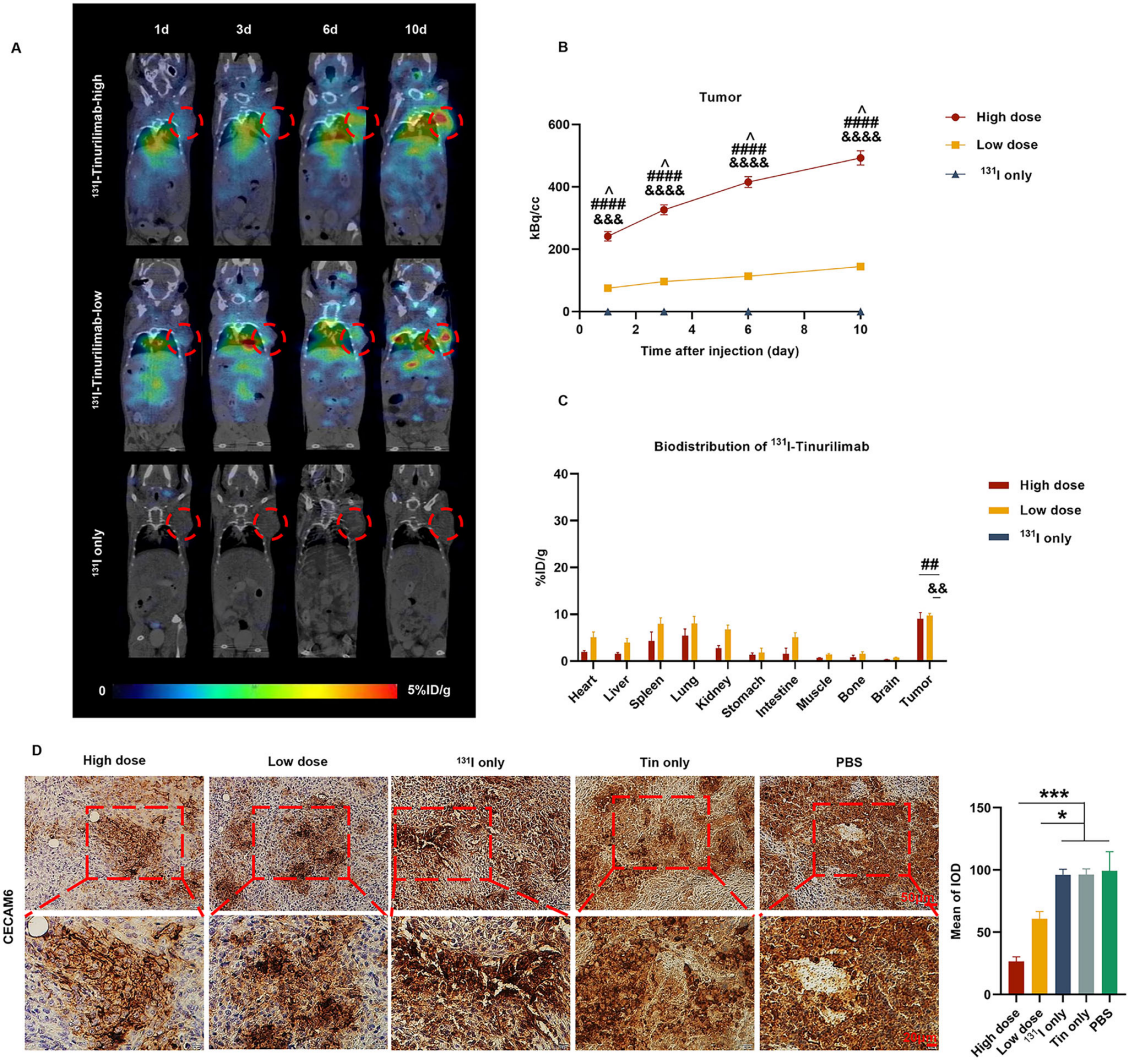
^131^I‐tinurilimab demonstrated specific and gradual uptake in CEACAM6‐positive tumor‐bearing mice. A) Representative SPECT images of ^131^I‐tinurilimab in A549 tumor‐bearing mice at different time points post‐injection. B) Quantitative analysis of the ROI derived from SPECT images in different dose of ^131^I‐tinurilimab treated A549 model mice. C) Ex vivo biodistribution of ^131^I‐tinurilimab in A549 model mice at 10 days post‐injection. D) Immunohistochemistry and quantitative analysis of CEACAM6 expression in A549 model mice after treated‐ or untreated ^131^I‐tinurilimab. Tin: Tinurilimab. All data are expressed as mean ± SEM. ^*^ *p* < 0.05, ^***^ *p* < 0.001. ^##^ *p* < 0.01, ^####^ *p* < 0.0001, High dose vs ^131^I only. ^&&^ *p* < 0.01, ^&&&^ *p* < 0.001, ^&&&&^ *p* < 0.0001, Low dose vs ^131^I only. ^^^ *p* < 0.05, High dose vs Low dose. *n* = 8 for each group.

To further substantiate the potential of ^131^I‐tinurilimab as a radioimmunotherapy in vivo, various doses of ^131^I‐tinurilimab (high dose of 16.5 or low dose of 5.5 MBq) were used in A549 xenografts for monitoring its targeting ability and biodistribution in vivo subsequent to a single treatment. The SPECT/CT analysis revealed that the uptake of either high or low doses of ^131^I‐tinurilimab in A549 xenografts exhibited a gradual increase from day 1 to day 10 after the treatment, in contrast to mice treated with free ^131^I (**Figure** **6**A). After ROI analysis, the radiation dose steadily accumulated starting from the first day of treatment and attained 492.89 ± 23.35 KBq cc^−1^ by the 10th day in mice treated with a high dose of ^131^I‐tinurilimab, which was significantly higher than the 144.10 ± 9.46 KBq cc^−1^ in mice subjected to the low‐dose treatment (Figure 6B). Meanwhile, no radiation dose was discerned in the mice treated with free ^131^I. And the distribution half‐life and clearance half‐life of ^131^I‐tinurilimab were 0.26 and 18.73 h, separately (Figure S5A, Supporting Information). This result revealed that the specific uptake of ^131^I‐tinurilimab in A549 xenografts and its accumulation within the tumor in an irradiation dose‐dependent manner were conducive to the efficacy of radioimmunotherapy.

The further ex vivo biodistribution showed that ^131^I‐tinurilimab was accumulated predominantly in tumor (9.01 ± 0.62% ID g^−1^ for the high‐dose treatment or 9.76 ± 0.21% ID g^−1^ for the low‐dose treatment) at the 10th day after treatment, indicating its potential therapeutic efficacy (Figure 6C). Subsequently, ^131^I‐tinurilimab was accumulated in the spleen, lung, and kidney. In addition, the immunohistochemical analysis showed that the expression of CEACAM6 was significantly decreased in high‐dose of ^131^I‐tinurilimab treatment mice compared with mice treated with free ^131^I (Figure 6D), which suggesting that the gradual escalation of ^131^I‐tinurilimab uptake by tumors was ascribable to its specific targeting and immunoreactivity, which ultimately accumulate in the tumor to eliminate cells, thereby reducing the expression of CEACAM6. All the in vivo results indicated that ^131^I‐tinurilimab was gradually accumulated in CEAMCA6‐positive xenografts and efficiently eliminated CEACAM6‐positive tumor cells.

### 2.7 ^131^I‐Tinurilimab Administration Significantly Inhibited the Growth of CEACAM6‐Positive Xenografts

**Figure 7:**
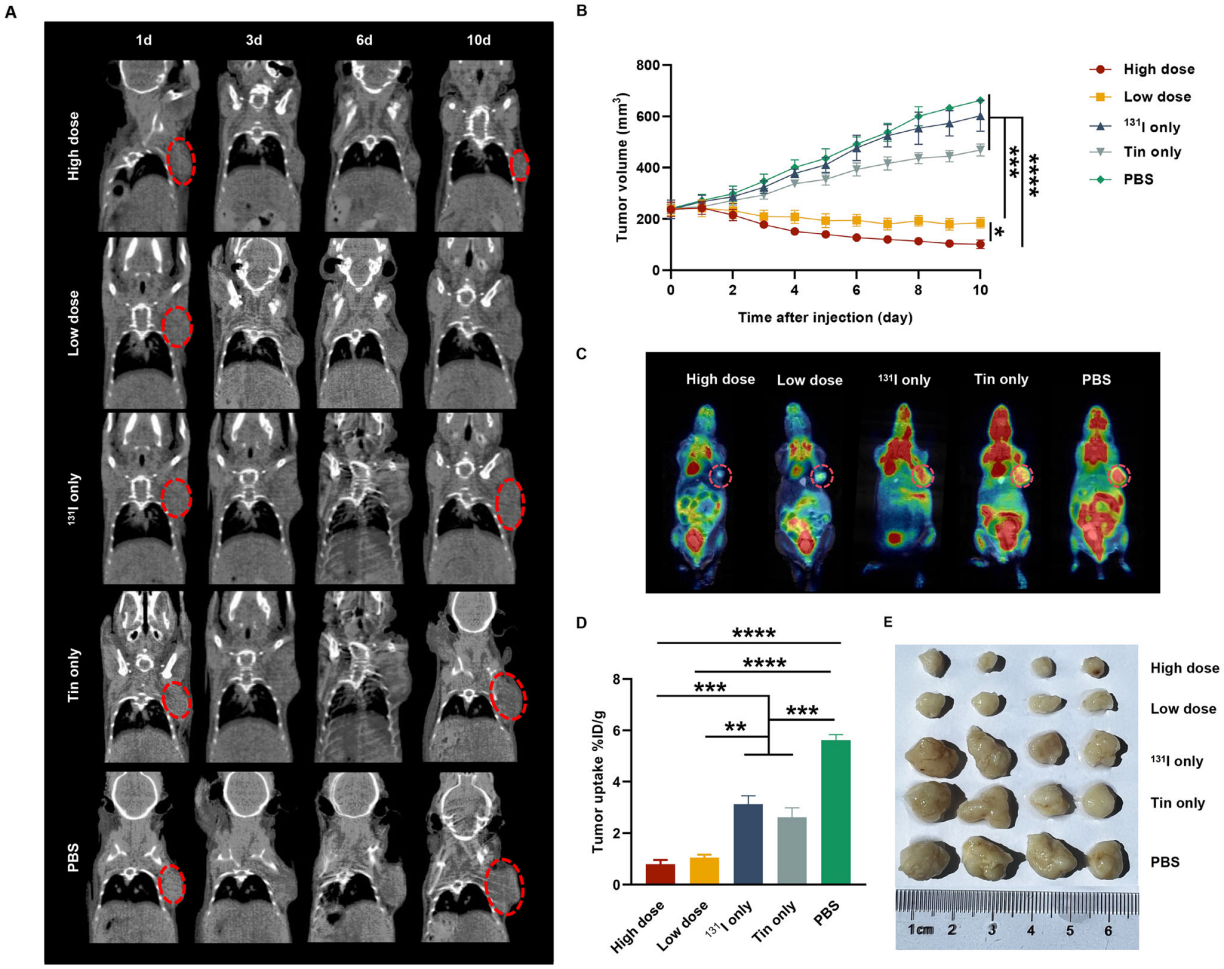
^131^I‐tinurilimab administration significantly suppressed tumor growth in CEACAM6‐positive tumor‐bearing mice. A) Representative microCT images of tumor size form 1th to 10th day in A549 model mice after ^131^I‐tinurilimab treatment. B) Tumor volume of different treatment groups in A549 model mice. C,D) Representative PET images and quantitative uptake analysis of ^18^F‐FDG in A549 model mice after ^131^I‐tinurilimab treatment. E) Ex vivo analysis of tumor size in A549 model mice after ^131^I‐tinurilimab treatment. Tin: Tinurilimab. All data are expressed as mean ± SEM. ^*^ *p* < 0.05, ^**^ *p* < 0.01, ^***^ *p* < 0.001, ^****^ *p* < 0.0001. *n* = 8 for each group.

After confirmed that ^131^I‐tinurilimab showed a potential therapeutic ability in CEACAM6‐positive xenografts, the treatments of PBS, tinurilimab, and free ^131^I were employed to compare the therapeutic effect with ^131^I‐tinurilimab in A549 xenografts. The microCT showed that ^131^I‐tinurilimab treatment significantly decreased tumor volume of A549 xenografts compared with PBS, tinurilimab or free ^131^I treated mice on the 10th day after injection (**Figure** **7**A). And high dose of ^131^I‐tinurilimab treatment showed a significantly decreased tumor volume compared with low dose of ^131^I‐tinurilimab treatment, suggesting that ^131^I‐tinurilimab eliminated tumor cells in a dose‐dependent fashion (Figure 7B). In order to further monitor the effect of ^131^I‐tinurilimab treatment, the ^18^F‐FDG PET was used for monitor the malignancy of the tumor in A549 xenografts mice. As shown in Figure 7C,D, the uptake of ^18^F‐FDG was significantly decreased in ^131^I‐tinurilimab treated A549 xenografts mice compared with PBS, tinurilimab or free ^131^I treated mice. In addition, ex vivo measurements of tumor size further redemonstrated that ^131^I‐tinurilimab treatment markedly reduced the size of A549 xenografts (Figure 7E). All these results revealed that ^131^I‐tinurilimab has an excellent performance in inhibiting the growth of CEACAM6‐positive xenografts, reflecting its potential therapeutic ability in LUAD or malignant pulmonary nodules. However, a gradual drop of body weight was observed in ^131^I‐tinurilimab treated groups, especially high dose of ^131^I‐tinurilimab treatment, mainly due to the relatively high radiation dose received. The weight drop was a bit better in low dose of ^131^I‐tinurilimab treatment groups after 10 days following the initiation of treatment (Figure S5B, Supporting Information). Notably, when survival curves were plotted, no significant changes in body weight were observed after a certain number of treatment days, suggesting the absence of severe toxic effects (Figure S6A, Supporting Information). This indicates that the treatments did not lead to substantial long‐term toxicity in the animals, supporting the safety profile of ^131^I‐tinurilimab. The two control groups, PBS and ^131^I only, demonstrated unrestrained tumor growth, with median survival times of 27 and 30 d, respectively. Interestingly, the data suggest that there is no direct relationship between survival time and tumor volume in the treatment groups. (Figure S6B, Supporting Information). These results underscore the therapeutic potential of ^131^I‐tinurilimab as a radiopharmaceutical.

### 2.8 ^131^I‐Tinurilimab Administration Enhanced DNA Damage in CEACAM6‐Positive Xenograft Tumor Cells

**Figure 8:**
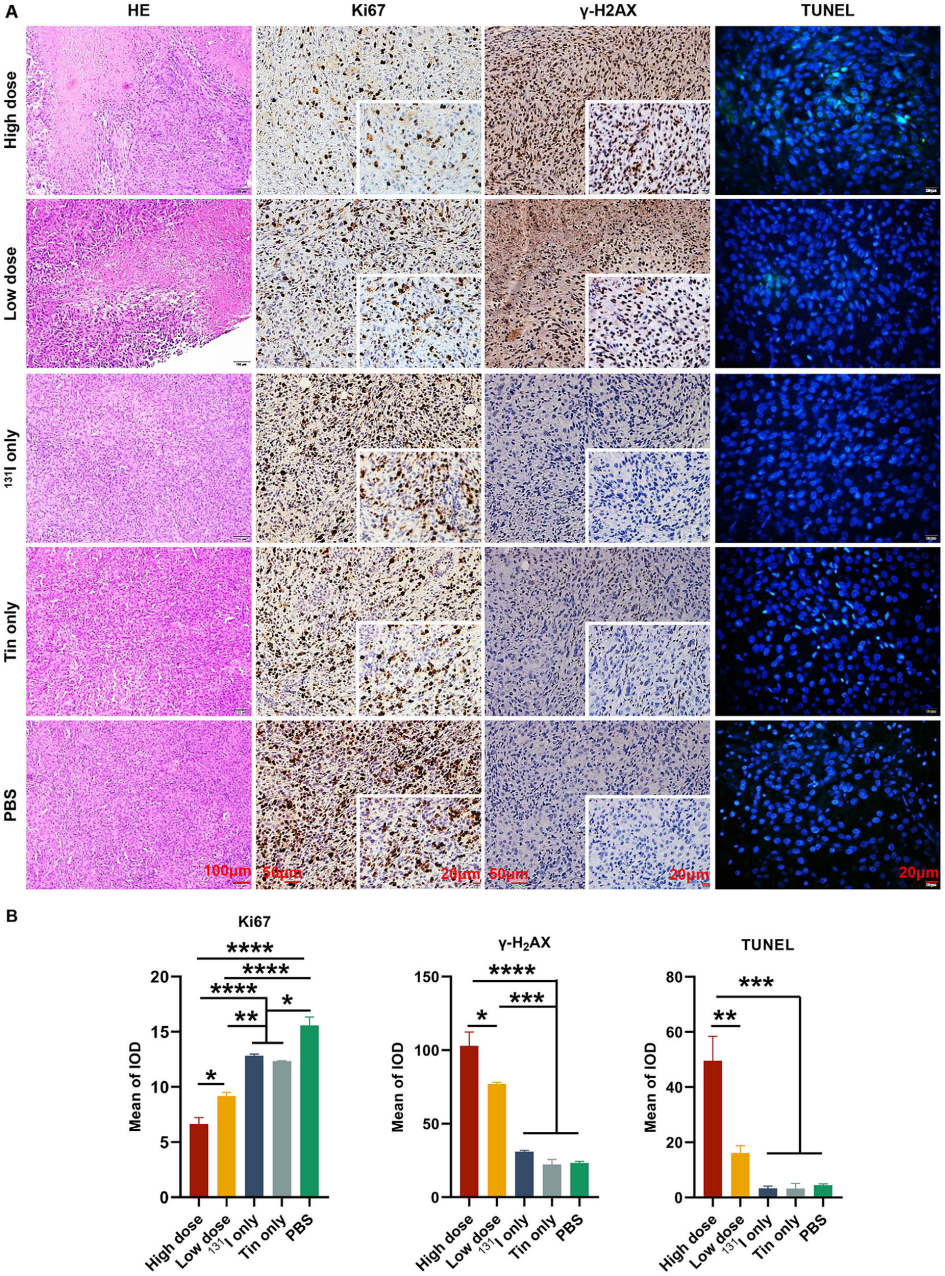
^131^I‐tinurilimab administration induced DNA damage, necrosis and apoptosis in CEACAM6‐positive tumor. A) Representative images of HE staining, immunohistochemistry (Ki67 and γ‐H2AX), and immunofluorescence (TUNEL) in A549 tumor after ^131^I‐tinurilimab treatment. B) Quantitative analysis the expression and intensity of Ki67, γ‐H2AX, and TUNEL‐positive area in ^131^I‐tinurilimab treated A549 tumor. Tin: Tinurilimab. All data are expressed as mean ± SEM. ^*^ *p* < 0.05, ^**^ *p* < 0.01, ^***^ *p* < 0.001, ^****^ *p* < 0.0001. *n* = 8 for each group.

Since the treatment of ^131^I‐tinurilimab conspicuously diminished the size of tumor, pathological analysis was used to further validate its therapeutic efficacy. The HE staining proved that ^131^I‐tinurilimab obviously increased necrosis in A549 xenografts compared with PBS, tinurilimab or free ^131^I treated mice, and high dose of ^131^I‐tinurilimab showed a more necrosis compared with low dose treatment (**Figure** **8**A). Radioimmunotherapy predominantly eliminates tumor cells by inducing DNA damage via the radioactivity of the conjugated nuclides. Immunohistochemical analysis showed that ^131^I‐tinurilimab significantly increased expression of γ‐H2AX in A549 xenografts compared with PBS, Tinurilimab or free ^131^I treated mice on the 10th day after injection. Furthermore, immunofluorescence analysis demonstrated that ^131^I‐tinurilimab significantly increased DNA damage‐induced apoptotic loci via TUNEL staining (Figure 8B). Additionally, ^131^I‐tinurilimab administration significantly suppressed cell proliferation in A549 xenografts as reflected by decreased expression of Ki67. All the results revealed that ^131^I‐tinurilimab was potentially capable of inhibiting the proliferation and deterioration of CEACAM6‐positive xenograft tumor cells through promoting DNA damage and inducing apoptosis.

## 3 Discussion

The early identification and treatment of malignant pulmonary nodules represent the most efficacious approaches of reducing the mortality of lung cancer, specifically LUAD. Considering the deficiency of LDCT in the discrimination between benign and malignant pulmonary nodules in a multitude of uncertain instances^[^ ^8^ ^]^ or still imposed excessive medical and psychological burdens,^[^ ^27^, ^28^ ^] 18^F‐FDG has been clinically advocated for the auxiliary diagnosis of malignant pulmonary nodules.^[^ ^29^ ^]^ Nevertheless, the specificity of ^18^F‐FDG may also be attenuated on account of inflammation, infection, and other factors.^[^ ^11^ ^]^ Furthermore, lobectomy and lymph node dissection are prevalently utilized in the clinical management of malignant pulmonary nodules, with the operative mortality rate ≈1 to 4%.^[^ ^17^ ^]^ Thus, novel management strategies still require further development. To compensate for the specificity of ^18^F‐FDG in the identification of malignant pulmonary nodules, machine learning was used to identify the specific biomarker between benign nodules and malignant tumors. Subsequently, PET imaging was employed to ascertain its potential in the identification of malignant lung nodules. Ultimately, therapeutic nuclide ^131^I was combined to explore the feasibility of targeting this biomarker for the theranostic of early LUAD. In this study, CEACAM6 was unambiguously recognized as the most preferable specific biomarker for discriminating benign nodules from LUAD, and its expression level was conspicuously correlated with the advancement of malignant nodules. The specificity and imaging performance of targeting CEACAM6 were notably superior to that of ^18^F‐FDG in PET imaging of distinguishing malignant pulmonary nodules. Additionally, radionuclide‐labeled CEACAM6 antibody (^131^I‐tinurilimab) was demonstrably capable of significantly inhibiting tumor growth in CEACAM6‐positive xenograft tumor models. Histological analysis unveiled the restricted toxicity of ^131^I‐tinurilimab, affirming that radioimmunotherapy targeting CEACAM6 constitutes an efficacious theranostic approach for malignant pulmonary nodules or early‐stage LUAD.

The investigation into non‐invasive and precise diagnosis of pulmonary nodules has advanced in recent years,^[^ ^30^ ^]^ whereas no regulatory approved and widely adopted non‐invasive methods are available for the effective identification of malignant nodules, except for ^18^F‐FDG PET. The majority of these studies have centered on blood biomarkers, such as plasma proteins (LG3BP and C163A),^[^ ^31^ ^]^ plasma cell‐free DNA (cfDNA) methylation,^[^ ^32^ ^]^ etc. Nevertheless, these non‐invasive blood biomarkers still entail certain specific drawbacks. For example, LG3BP and C163A demonstrate low specificity for larger nodules,^[^ ^33^ ^]^ the sensitivity of cfDNA in plasma is influenced by multiple factors and the specificity remains inadequate.^[^ ^34^ ^]^ Compared to widely employed biomarkers such as carcinoembryonic antigen (CEA) and cytokeratin 19 fragment (CYFRA 21–1), a study involving a cohort of 300 serum samples demonstrated that serum CEA levels for diagnosing lung adenocarcinoma (LUAD) yielded an area under the curve (AUC) of 0.67, with a sensitivity of 24.6% at a specificity of 97.6%. Furthermore, there was no statistically significant difference in serum CYFRA 21–1 levels between the LUAD group and the benign nodules group.^[^ ^35^ ^]^ While, in this study, the AUC for CEACAM6 in differentiating between benign nodules and LUAD was 0.77, with a sensitivity of 71% % at a specificity of 82%, which proved that it can be a promising biomarker for identification of malignant pulmonary nodules at early stage of LUAD. PET can visually and objectively assess the benign and malignant nodules, featuring non‐invasive, quantitative, real‐time, specific, and high sensitivity. Thus, the PET imaging specific biomarker will furnish direct and objective clinical diagnostic findings for the identification of malignant pulmonary nodules. CEACAM6, identified as most privileged specific biomarker for distinguishing benign nodules from LUAD, showed a positive correlation with progression of malignant nodules and highly specific in delineating malignant nodules with a background target ratio of 9.93 (considerably superior to ^18^F‐FDG). The preclinical mouse model of malignant pulmonary nodules attested that the integration of specific biomarker and targeted PET imaging constitutes a promising solution to mitigate the deficiency of ^18^F‐FDG in the clinical identification of malignant pulmonary nodules. Additionally, animal models have shown that PET imaging targeting CEACAM6 is capable of identifying malignant nodules ≈2 mm (1.76 mm). This also substantiated that PET imaging employing targeted specific biomarker may further redress the sensitivity shortfall induced by conventional ^18^F‐FDG PET (failure to identify nodules smaller than 8 mm).

Integrated radionuclide therapy constitutes a revolutionary cancer diagnosis and treatment paradigm that seamlessly transitions from diagnosis to treatment through the combination of diagnostic and therapeutic radionuclides with targeted ligands, such as ^68^Ga‐PSMA‐11/^177^Lu‐PSMA‐617 for metastatic castration‐resistant prostate cancer,^[^ ^36^ ^] 89^Zr/^177^Lu‐aflibercept for breast cancer.^[^ ^37^ ^]^ After confirmed the crucial significance of CEACAM6 in the identification of malignant pulmonary nodules, targeted radioimmunotherapy was implemented to accomplish an integrated diagnosis and treatment approach in order to preclude the sequelae induced by invasive surgery. In this study, ^89^Zr‐Df‐Tinurilimab showed a high tumor uptake at around 17% ID g^−1^ and low uptake in other nontarget organs in CEACAM6‐positive models, providing excellent tumor contrast. Regarding differential diagnosis, the distinct performance and resolution of PET imaging disclosed that ^89^Zr‐Df‐tinurilimab was ≈4 times superior to ^18^F‐FDG in differentiating malignant pulmonary nodules, confirming its outstanding diagnostic performance in the identification of malignant pulmonary nodules. In therapeutic aspect, CEACAM6, functioning as an immunosuppressive modulator, and its targeted antibody tinurilimab, have been utilized in preclinical studies for the treatment of solid tumors.^[^ ^20^ ^]^ The high uptake and excellent tumor contrast of ^89^Zr‐Df‐tinurilimab indicated that tinurilimab was suitable for conjugating therapeutic nuclides in the context of radioimmunotherapy. Among a plethora of therapeutic nuclides, ^131^I possesses a prolonged half‐life, is appropriate for SPECT diagnosis, and its labeling approach is straightforward, without influencing the structure and activity of antibodies.^[^ ^38^, ^39^ ^]^ In our study, the affinity of tinurilimab remained high even after ^131^I labeling (Kd = 0.3 nm), and ^131^I‐tinurilimab was progressively and significantly accumulated in CEACAM6‐positive tumor after once administration. In addition, both high or low dose of ^131^I‐tinurilimab administration significantly suppressed malignancy of tumor reflecting by decreased tumor size and glycometabolism (limited uptake of ^18^F‐FDG). Pathological and immunohistochemical analysis confirmed that ^131^I‐tinurilimab induced DNA damage and apoptosis, ultimately giving rise to the necrosis of tumor tissues. These findings minimally indicated that ^131^I‐tinurilimab holds therapeutic advantages for CEACAM6‐positive tumors and could potentially contribute to the noninvasive ablation of CEACAM6‐positive malignant pulmonary nodules in the future clinical practice.

In addition to the promising diagnostic and therapeutic potential of CEACAM6‐targeted radioimmunotherapy, several factors enhance its feasibility and clinical value. First, the scalability of this approach is supported by the widespread availability and low cost of ^131^I, a radionuclide with a long history of clinical use.^[^ ^40^ ^]^ This, combined with the commercial availability of tinurilimab, a mature antibody suitable for clinical studies (NCT03596372), ensures that the production and application of ^131^I‐tinurilimab can be easily scaled for broader clinical use. Furthermore, the relatively simple ^131^I labeling process makes this therapy more accessible for large‐scale implementation. From a regulatory perspective, ^131^I is already well‐established in clinical practice (such as ^131^I‐omburtamab in treatment of CNS malignancies),^[^ ^41^ ^]^ and the combination of diagnostic PET imaging with CEACAM6‐targeting and therapeutic radionuclides aligns with current trends in theranostic oncology. This can facilitate the approval process, particularly as the specificity and low toxicity of ^131^I‐tinurilimab in targeting CEACAM6‐positive tumors support its safety profile. In terms of cost‐effectiveness, ^131^I's low cost, combined with the availability of tinurilimab, makes this approach an economically viable option for treating LUAD. Moreover, integrating diagnostic imaging with therapeutic treatment in a single modality reduces the need for multiple tests and procedures, further improving the cost efficiency. Taken together, these factors make CEACAM6‐targeted ^131^I‐tinurilimab radioimmunotherapy a scalable, cost‐effective, and clinically promising approach for the diagnosis and treatment of malignant pulmonary nodules in LUAD.

The potential toxicity of radioactivity serves as the predominant factor impinging upon radioimmunotherapy.^[^ ^42^ ^]^ Due to the lengthy half‐life of tinurilimab, the metabolism in vivo was rather slow, thereby prolonging the duration of radiation exposure in vivo. It is incontrovertible that the ^131^I‐tinurilimab treatment induced weight loss in A549 tumor‐bearing mice ten days following the initiation of treatment. Nevertheless, the histopathological examination disclosed that the slight deformation of hepatic sinus was merely found in liver (the top uptake of ^131^I‐tinurilimab), and the other major organs (heart, lung, pancreas, and kidney) exhibited no conspicuous toxic side effects. Previous study also proved that radioimmunotherapy will decrease the body weight of mice with xenografted tumors,^[^ ^23^ ^]^ which could potentially arise from the distinct susceptibilities of diverse types of mice to irradiation.^[^ ^43^ ^]^ In addition, a considerable number of studies have indicated that ^131^I‐labeled antibodies utilized for the treatment of xenograft tumors have been proven to be safe or tolerable in terms of toxic side effects. For instance, 74 MBq of ^131^I‐antiEGFR‐BSA‐PCL treatment showed no difference in body weight and other obvious side‐effects in mouse model of colorectal cancer,^[^ ^44^ ^]^ the treatment of 375 and 1125 uCi ^131^I‐CAb1 F(ab')2 in mouse model of human colon cancer xenografts was proved to be safe and effective,^[^ ^45^ ^]^ and the intravenous injection of ^131^I‐radiolabeled MoAb (0.125‐1 mCi) in BALB/c nude mice of human neuroblastoma (NB) xenografts was proved to be tolerable toxicities.^[^ ^46^ ^]^ Thus, these results suggested that the dose of ^131^I‐tinurilimab (150 uCi/5.55 MBq or 450 uCi/16.5 MBq) would have limited toxicity and accepted biosafety in treatment of CEACAM6‐positive malignant pulmonary nodules or LUAD. Furthermore, over an extended period exceeding the initial 10 day observation window post‐treatment initiation, we observed no significant alterations in the body weight of the mice relative to baseline. This finding further supports the conclusion that ^131^I‐tinurilimab treatment lacks toxic side effects.

It is notable that although preclinical data studies have indicated that ^89^Zr/^131^I labeled tinurilimab possesses potential application significance in the theranostic of malignant pulmonary nodules, it also entails certain disadvantages. For example, the diagnostic time of ^89^Zr‐Df‐tinurilimab PET imaging was prolonged, and the β–ray treatment of ^131^I‐tinurilimab treatment was less effective and safety than that of α–ray particle treatment (^212^Pb and ^225^Ac).^[^ ^47^, ^48^ ^]^ To address this deficiency, the structural optimization of antibodies (such as nano–antibodies) and the optimization of diagnostic nuclides (such as ^68^Ga labeling) can be implemented, such as ^68^Ga/^255^AC–DOTA–4AH29 for theranostic of FAP‐positive tumor.^[^ ^49^ ^]^ These modifications are also ongoing in the subsequent experiments.

## 4 Conclusion

This study demonstrated that CEACAM6 can function as a target for the theranostics of malignant pulmonary nodules, warranting further exploration in the clinical domain. Highly specific and distinctly differentiated malignant pulmonary nodules, along with significant tumor suppression, were observed in radiolabeled tinurilimab. ^131^I‐tinurilimab was observed negligible toxicity in the histological analysis. These results suggested that radiation therapy targeting CEACAM6 will be widely used in clinical CEACAM6‐positive tumors.

## 5 Experimental Section

### Materials

The monoclonal antibody of tinurilimab with a purity greater than 98% was purchased from Absin (Shanghai, China), and *p*‐Isothiocyanatobenzyldesferrioxamine (*p*‐NCS‐Bn‐Df) was obtained from Macrocyclics (TX, USA). PD–10 columns were acquired from GE Healthcare, and the Detergent Compatible Bradford Protein Assay Kit was sourced from Beyotime Biotechnology. Zeba Protein Desalting Spin Columns (MW cutoff 7 kDa) were purchased from Millipore (Thermo Scientific, USA).

### Machine Learning (ML)

The proteomic data of benign pulmonary nodules and LUAD acquired from previous study^[^ ^13^ ^]^ was used to ML analysis. The differentially analysis of proteins expression was performed by Perseus software with a cut off value of *p* < 0.05, Fold change ≥ 1, and the obtained upregulated proteins were filtered out for subsequent analysis. The Venny analysis was used for selecting LUAD specific proteins derived from differential analysis and then the obtained proteins was enriched for cell component enrichment analysis. After that, the cell‐surface proteins were acquired for ML performance including LASSO and random forest. Shortly, the optimum penalty parameter *λ* was identified through the application of LASSO with minimum binomial deviation, and subsequently, the biomarker proteins were acquired in accordance with *λ*. The R package of “Random Forest” was used to identify the proteins with the smallest error and to rank the importance of the proteins. Finally, the biomarker proteins were filtered out based on the Venn analysis of the shared biomarkers among those machine learning‐based classification algorithms. The diagnostic potential of hub biomarkers was assessed via ROC curve analysis and AUC calculation with the “pROC” package in Rstudio.

### Western Blotting

Human hepatocarcinoma cells (Huh7) and human LUAD cell lines, including PC9, NCI–H1975, Calu3, and A549, were obtained from the American Type Culture Collection (ATCC) via the Shanghai Stem Cell Bank. All cell lines were cultured under standard conditions of 37 °C in a humidified atmosphere with 5% CO_2_, using DMEM (high‐glucose) supplemented with 10% fetal bovine serum and 1% penicillin‐streptomycin.

The western blot analysis was performed according to a standard method. Briefly, after extracting and quantifying the proteins from A549, Huh7, PC9, and NCI‐H1975, the sample was loaded onto an SDS–PAGE gel for electrophoresis. Then, the proteins were transferred to a PVDF membrane for antibody incubation. After blocking, the membrane was incubated overnight at 4 °C with rabbit monoclonal anti‐human CEACAM6 (1:1000, Abcam) and rabbit anti‐human β–tubulin (1:2000, Proteintech) antibodies. Finally, an HRP‐labeled goat anti‐rabbit IgG (H+L) secondary antibody was applied, and the membrane was scanned and analyzed using the BeyoECL Plus Western Blotting Detection System (Beyotime). Grayscale values were quantified using ImageJ software.

### Evaluation of CECAM6 for Diagnosing LUAD Progression using ^89^Zr‐Labeled Tinurilimab—Tinurilimab Conjugation and Flow Cytometry

Tinurilimab was prepared for radiolabeling through conjugation with *p*‐SCN‐Bn‐deferoxamine (Df, Macrocyclics) at a mole ratio of 1:10 (antibody: chelator) in a solution adjusted to pH 9.0 using 0.1 m Na_2_CO_3_ for 90 min, following a standard protocol. Excess chelator was removed using a Zeba Protein Desalting Spin Column. The final concentration of tinurilimab was determined using the Detergent Compatible Bradford Protein Assay Kit.

Direct methods were employed to assess cellular affinity according to established protocols. Sulfo‐Cyanine5 succinimidyl ester (Cy5 NHS Ester, MedChemExpress) was conjugated to the antibody, purified, and utilized to process Cy5‐conjugated tinurilimab or Df‐tinurilimab. Flow cytometry was conducted using a BD FACSCalibur Flow Cytometer (BD Biosciences), with FlowJo software employed to analyze mean fluorescence intensities.

### Evaluation of CECAM6 for Diagnosing LUAD Progression Using ^89^Zr‐Labeled Tinurilimab—Radiolabeling and In Vitro Stability Evaluation

For ^89^Zr labeling, ^89^Zr‐oxalate in 4‐(2‐hydroxyethyl)‐1‐piperazineethanesulfonic acid (HEPES) buffer was added to the Na_2_CO_3_ solution containing Df‐tinurilimab (pH 7.0) and mixed for 10 min at 37 °C. Radiolabeled products were purified using PD‐10 columns with PBS. ^89^Zr‐oxalate was purchased from Yantai Dongcheng Pharmaceutical Group Co., Ltd. The radiochemical purity and stability of ^89^Zr‐labeled tinurilimab were analyzed using radio thin layer chromatography (TLC, BioScan) with 0.5 m citrate buffer (pH 5) as the mobile phase. Stability assessments were conducted by incubating the radiotracers in PBS or FBS at room temperature for up to 10 days.

### Evaluation of CECAM6 for Diagnosing LUAD Progression using ^89^Zr‐Labeled Tinurilimab—Cellular Uptake

Cell binding assays were performed by incubating various cell lines with 37 KBq of ^89^Zr‐labeled tinurilimab at 37 °C for differing durations. To assess binding specificity, a blocking group was co‐incubated with a 100‐fold excess of unlabeled tinurilimab (34 µg tube^−1^). Unbound radiotracer was removed by washing the cells twice with ice‐cold PBS. Radioactivity in the fractions was measured using a γ‐counter (Perkin Elmer), with uptake expressed as a percentage of the radioactive dose per 10^5^ cells (% AD/10^5^ cells).

### Evaluation of CECAM6 for Diagnosing LUAD Progression Using ^89^Zr‐Labeled Tinurilimab—Immunoreactivity Analysis of the Radioimmunoconjugate

The immunoreactive fraction of ^89^Zr‐labeled tinurilimab binding to CEACAM6 was determined by extrapolating binding to infinite antigen excess using a Lindmo assay. A serial dilution of A549 cells (1 × 10^6^ to 128 × 10^6^ cells mL^−1^) was incubated with 3.7 KBq of the radiolabeled antibody at 4 °C for 2 h. The resulting cell pellets were measured using a γ‐counter to calculate the maximum binding ability (Bmax) and affinity constant (Ka).

### Evaluation of CECAM6 for Diagnosing LUAD Progression using ^89^Zr‐Labeled Tinurilimab—PET Imaging of ^89^Zr‐Labeled Tinurilimab

Six‐week‐old female nude mice (Cavens Animal Co. Ltd.) were implanted with cells (A549, Huh7, PC9, and Calu3) at a concentration of 5 × 10^6^ cells mL^−1^ to establish subcutaneous tumor models. Treatment commenced once the tumors reached a diameter of 5–10 mm. Mice were monitored every other day for body weight and overall health. PET imaging was conducted on a micro‐PET scanner (Inveon, Siemens) following intravenous injection of ^89^Zr‐Df‐tinurilimab (≈0.55 MBq per mouse, *n* = 5). Blocking experiments included pre‐injecting a 100‐fold excess of tinurilimab (0.5 mg per mouse) one hour before the radiotracer to occlude receptor binding. Static PET images were acquired over a 10 min period at 2, 6, 12, 24, 48, 72, and 120 h post‐injection. Quantitative analysis involved defining regions of interest (ROI) at the tumor and heart, processed using ASIPro software.

All animal experiments were conducted in accordance with the Laboratory Animal Guidelines and received approval from the ethics committee of the Jiangsu Institute of Nuclear Medicine, in compliance with national laws and regulations (JSINM‐2024‐110 and JSINM‐2023‐079).

### Evaluation of CECAM6 for Diagnosing LUAD Progression using ^89^Zr‐Labeled Tinurilimab—In Vivo PET/MR Imaging in Urethane‐Induced LUAD Model

According to the previous study of constructing urethane‐induced LUAD model,^[^ ^50^ ^]^ 10‐week‐old A/J mice (procured from Cavens Animal Co. Ltd.) were received a single intraperitoneal injection of urethane (100 mg/100 g, Sigma Aldrich). Then, all the mice were subjected to micro‐computed tomography (micro–CT, Albira SI, Bruker) imaging at 8‐ and 16‐weeks post‐urethane administration.

Mice were injected with ^89^Zr‐Df‐tinurilimab (≈0.55 MBq per mouse, *n* = 4) via the tail vein and anesthetized using isoflurane for PET/MR imaging at 24, 48, or 72 h post‐injection. Scans were carried out on a PET/MR scanner (Bruker, 9.4T) with 20 min static PET/MR scans performed at each time point. Images were reconstructed using a 3D iterative ordered subset expectation maximization (3DOSEM) method. ROI were delineated with MRI guidance, and data were analyzed via PMOD 4.4 software, with radioactivity concentration expressed as % ID g^−1^.

### Evaluation of CECAM6 for Diagnosing LUAD Progression using ^89^Zr‐Labeled Tinurilimab—^18^F‐Fluorodeoxyglucose (^18^F‐FDG) PET/MR Imaging

^18^F‐FDG PET imaging was conducted at 8‐ and 16‐weeks after urethane administration to monitor the progression of LUAD. Mice were fasted for at least 12 h prior to imaging. Each mouse was anesthetized for a minimum of 50 min following injection of ^18^F‐FDG (≈3.7 MBq). A total of 20 million coincidence events were acquired per mouse. Quantitative analysis involved drawing regions of interest (ROIs) on the tumor regions. The ^18^F‐FDG was provided by the Affiliated Hospital of Jiangnan University.

### Evaluation of ^131^I‐Labeled Tinurilimab in CEACAM6‐Positive Tumors—Radiolabeling and In Vitro Stability Evaluation

For ^131^I labeling, Na^131^I was added to the reaction tube containing tinurilimab, followed by the addition of 20 µg of freshly prepared chloramine‐T (J&K) in phosphate–buffered saline (PBS), mixed for 15 min. Radiolabeled products were purified using PD‐10 columns with PBS. And Na^131^I was obtained from the Chengdu Gaotong Isotope Corporation (China Nuclear Group). The protocol of radiochemical purity and stability of ^131^I‐labeled tinurilimab was same with ^89^Zr‐labeled tinurilimab.

### Evaluation of ^131^I‐Labeled Tinurilimab in CEACAM6‐Positive Tumors—Cellular Uptake, Immunoreactivity and Internalization Analysis of ^131^I‐Labeled Tinurilimab

The protocol for cellular uptake and immunoreactivity analysis of ^131^I‐labeled tinurilimab was identical to that of ^89^Zr‐labeled tinurilimab, as detailed in the preceding section. For internalization analysis, an internalization assay was conducted following the methodology outlined with modifications. A total of 5 × 10^5^ cells were seeded in 12‐well plates and incubated overnight at 37 °C. After removing the media, fresh media containing ^131^I‐tinurilimab (37 kBq) was added, and plates were incubated at 37 °C for 0.5, 1, 2, 4, and 8 h. Surface‐bound radioactivity was removed by incubating the cells with 500 µL of 0.2 m glycine in 4 m urea at 37 °C for 5 min. Cells were then lysed with 1 m NaOH, dissociated from the wells, and transferred to microcentrifuge tubes. Both the surface‐bound and cell lysate fractions were counted using a γ‐counter.

### Evaluation of ^131^I‐Labeled Tinurilimab in CEACAM6‐Positive Tumors—In Vitro Cytotoxicity of ^131^I‐Tinurilimab

A549 cells were seeded in 96‐well plates at a density of 5 × 10^3^ cells per well. The following day, cells were treated with either ^131^I or ^131^I‐tinurilimab across a range of radioactivity levels (0.45 to 7.4 MBq) and co‐cultured for 72 h. Cell survival rates were assessed using the standard CCK‐8 method.

### Evaluation of ^131^I‐Labeled Tinurilimab in CEACAM6‐Positive Tumors—Therapeutic Administration and Monitoring

A single‐dose administration strategy was monitored over 10 d according to previous protocols. Treatment groups included high‐dose (50 µg, 16.5 MBq) and low‐dose (5.5 MBq) ^131^I‐tinurilimab, along with control groups of free ^131^I (16.5 MBq), tinurilimab (50 µg), and PBS. Following tail‐vein injection, tumor volume and body weight were recorded every other day. Tumor volume was calculated as 1/2 × length × width.^[^ ^2^ ^]^ Standardized tumor volume was derived by normalizing to initial volume, multiplied by 100, and expressed as a percentage. Standardized body weight was similarly calculated. ^18^F‐Fluorodeoxyglucose (^18^F‐FDG) PET imaging was employed to evaluate the malignancy of tumors following diverse treatment regimens. The treatment of the mice was carried out in accordance with humane and ethical standards for animal welfare.

### Evaluation of ^131^I‐Labeled Tinurilimab in CEACAM6‐Positive Tumors—SPECT/CT Imaging

Planar scans were obtained at 1, 3, 6, and 10 d after the injection of radioactive probes, included high‐dose and low‐dose ^131^I‐tinurilimab, ^131^I only, Tin only, and PBS. Mice were anesthetized and positioned supine during imaging, which lasted for 45 min using a single‐photon emission computed tomography/computed tomography (SPECT/CT) system (Albira SI, Bruker). The radioactivity concentration within the organs was calculated and expressed in KBq/cc.

### Pharmacokinetics Study

For the pharmacokinetics study of ^89^Zr‐ and ^131^I‐labeled tinurilimab, blood samples were collected from the tail vein of tumor‐bearing mice (*n* = 4) at various time points. Each blood sample was weighed, and its radioactivity was measured using a γ‐counter. Pharmacokinetic parameters were subsequently analyzed using the DAS2.1 software.

### Biodistribution

Ex vivo biodistribution studies were performed for both ^89^Zr‐ and ^131^I‐labeled probes following the final imaging time point in tumor‐bearing mice. After euthanizing the mice via CO_2_ asphyxiation, organs of interest were harvested and weighed. The radioactivities of these organs were measured using a γ‐counter, with the biodistribution results expressed as % ID/g.

### Histological Staining

Histological analyses of tissues included hematoxylin and eosin (H&E) staining, immunohistochemistry, and terminal deoxynucleotidyl transferase deoxyuridine triphosphate nick end labeling (TUNEL) staining. H&E staining was performed on tumor, heart, lung, liver, kidney, and small intestine tissues following standard protocols. Immunohistochemistry staining for CEACAM6 expression was conducted on A549 and Huh7 tumor tissues as well as on lung nodule tissues using a rabbit monoclonal anti‐human CEACAM6 antibody (1:2000) and a DAB Detection Polymer Kit (Gene Tech). Additionally, Ki67, γH2AX, and TUNEL assay were employed to evaluate apoptosis in tumor tissues from those experiment groups, following standard procedures. Tissue morphology and staining were documented, with image analysis performed using ImageJ software.

### Statistical Analysis

All quantitative data were presented as mean ± SEM. Statistical analysis was performed using either an ANOVA (equal variance) or a Welch's ANOVA (unequal variance) test followed by Bonferroni's post‐hoc test with GraphPad (Prism 8.0). Tumor growth volume and body weight comparisons among different therapeutic groups were performed using two‐way repeated measures ANOVA. For survival analysis, the time periods at risk for tumor volume exceeding 1500 mm^3^ or for death were calculated in days for each mouse. Events that did not result in tumor oversize or death were considered due to the termination of the observation period. Exploratory analyses indicated that the relationship between the type of therapy and the rate of tumor oversize remained consistent over time. Survival curves for different therapeutic groups were compared using the log‐rank test. Statistical significance was defined as *p* < 0.05.

## Conflict of Interest

The authors declare no conflict of interest.

## Author Contributions

C.C., K.Z., and J.W. contributed equally to this work. C.C., K.Z., and J.W. drafted the manuscript and figures. D.P., X.W., Y.X., J.Y., and Z.W. helped figure modification. M.Y. designed the experiments and revised the manuscript. All authors read and approved the final manuscript.

## Supporting information



Supporting Information

## Data Availability

The data that support the findings of this study are available from the corresponding author upon reasonable request.
