# Supplementary material for: A Novel Theranostic Strategy for Malignant Pulmonary Nodules by Targeted CECAM6 with 89Zr/131I‐Labeled Tinurilimab
Source: Adv Sci (Weinh). 2025 Apr 3;12(21):2415689. doi: 10.1002/advs.202415689 (PMC12140296; doi:10.1002/advs.202415689)
Supplement: Supplementary file 1 — Supporting Information [file ADVS-12-2415689-s001.docx]

**A novel theranostic strategy for malignant pulmonary nodules by targeted CECAM6 with ^89^Zr/^131^I-labeled Tinurilimab**

Chongyang Chen^#/1,2^, Keying Zhu^#/1,2^, Jing Wang^#/3^, Donghui Pan^1^, Xinyu Wang^1^, Yuping Xu^1^, Junjie Yan^1^, Lizhen Wang^1^, Min Yang^*/1,2^

**Materials and methods**

Small interfering RNA (siRNA) cellular transfection

A549 cells (2×10^5^ cells) were plated on 6-well plates for 24 h prior to siRNA transfection. CEACAM6 siRNA1 (sense, 5’-UCUUAUUUUAUGUAAAACGUU-3’; antisense, CGUUUUACAUAAAAUAAGAGA-3’), siRNA2 (sense, 5’-UAAAAUAGAAUAAAGAAUGAG-3’; antisense, 5’-CAUUCUUUAUUCUAUUUUAGU-3’), siRNA2 (sense, 5’- AAUCUUAAAGCAUUAGUGCUA-3’; antisense, 5’-GCACUAAUGCUUUAAGAUUUG-3’) and Lipo3000 transfection reagent (Thermo Fisher) were added, and then incubated for 48h. After siRNA transfection, A549 cells were used for western blots and wound healing assay.

Western blots

Cell lysates or immunoprecipitates were heated at 98 °C for 10 minutes. Proteins were separated using SDS-PAGE and transferred onto polyvinylidene fluoride (PVDF) membranes. The membranes were blocked with TBS-T containing 5% BSA for 2 hours at room temperature, followed by incubation with primary antibodies at the following dilutions: anti-FAK (Proteintech, 1:1000), anti-phospho-FAK (Tyr397) (Proteintech, 1:1000), anti-Src (STARTER, 1:1000), anti-phospho-Src (Tyr416) (STARTER, 1:1000), anti-CEACAM6 (Absin, 1:1000), and anti-GAPDH (Proteintech, 1:1000). Subsequently, HRP-conjugated goat anti-rabbit or anti-mouse IgG (H+L) secondary antibodies were applied. Membranes were scanned and analyzed using the BeyoECL Plus Western Blotting Detection System (Beyotime). Grayscale values were quantified with ImageJ software.

Wound healing assay

Wound images were captured at 0- and 24- hours post-scratching using a sterile 10 μL pipette. The extent of wound closure was quantified at four distinct positions using ImageJ software. Triplicate experiments were conducted to ensure reliable and consistent results.

**Supplementary Figures**

**Supplementary Figure 1. Silencing CEACAM6 expression inhibited both the Src/FAK signaling pathway and cell migration.**

**Supplementary Figure 2. Immunofluorescence analysis of CEACAM6 expression and determination of the labeling yield of ^89^Zr/^131^I labeled Tinurilimab.**

**Supplementary Figure 3. ^89^Zr-Df-Tinurilimab exhibited specific uptake in PC9 and Calu3 tumor-bearing mice.**

**Supplementary Figure 4. Representative images of HE staining of A549 model mice after ^89^Zr-Df-Tinurilimab injection.**

**Supplementary Figure 5. Administration of ^131^I-Tinurilimab resulted in a minor toxic effect in A549 model mice on the 10th day following initial treatment.**

**Supplementary Figure 6. Antitumor treatment study of ^131^I-Tinurilimab in A549 tumor-bearing mice.**


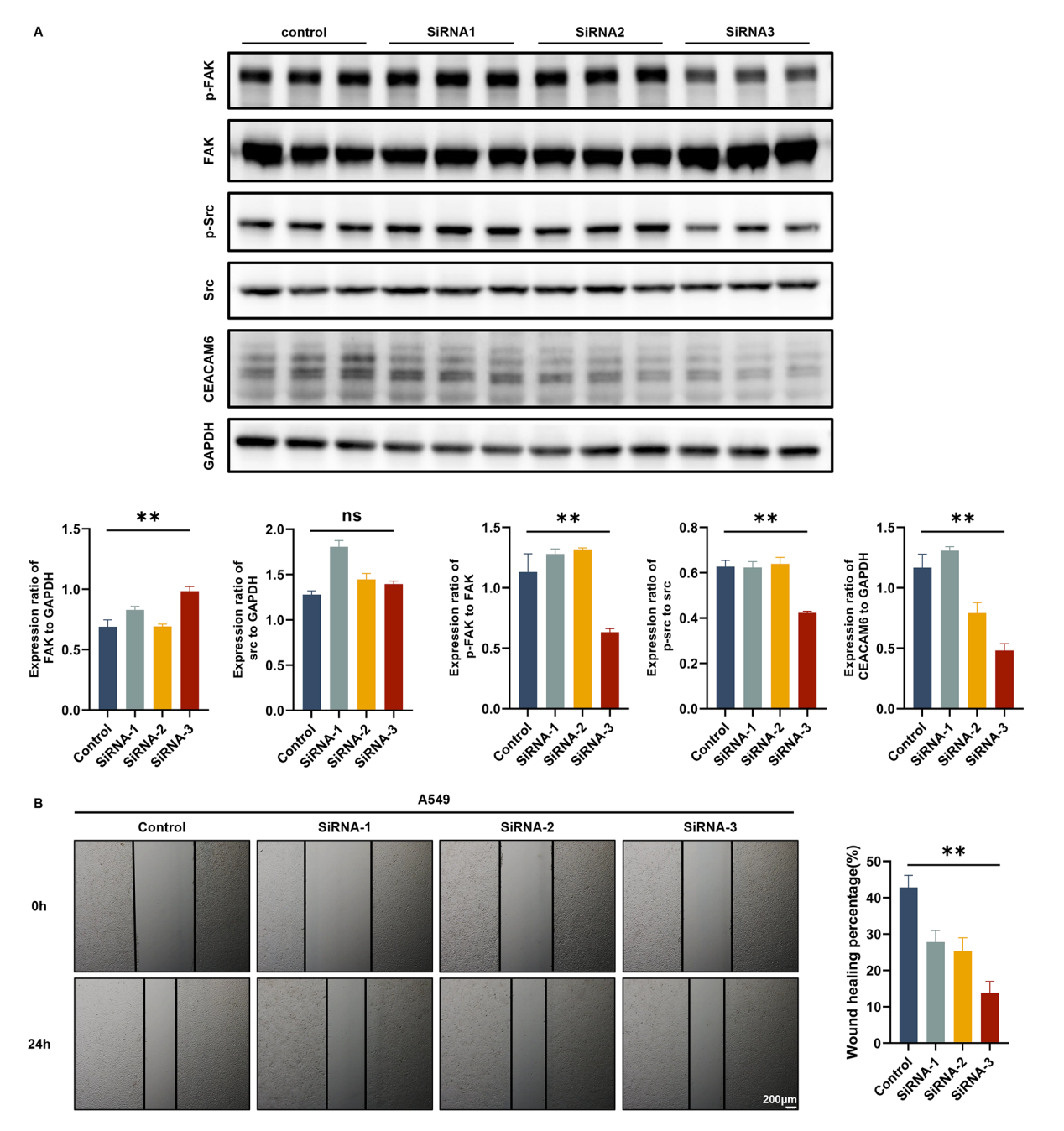


**Supplementary Figure 1. Silencing CEACAM6 expression inhibited both the Src/FAK signaling pathway and cell migration.**

(A) Western blots analysis and quantified proteins expression of Src/FAK signaling pathway (p-Src, Src, p-FAK, FAK) in CEACAM6 knockdown A549 cells. (B) Wound healing assay was performed on CEACAM6 knockdown A549 cells. Relative scratch covered area was quantified by Image J from four different areas. All data are expressed as mean ± SEM. **p < 0.01, siRNA3 versus Control. n = 3 for each group.

**
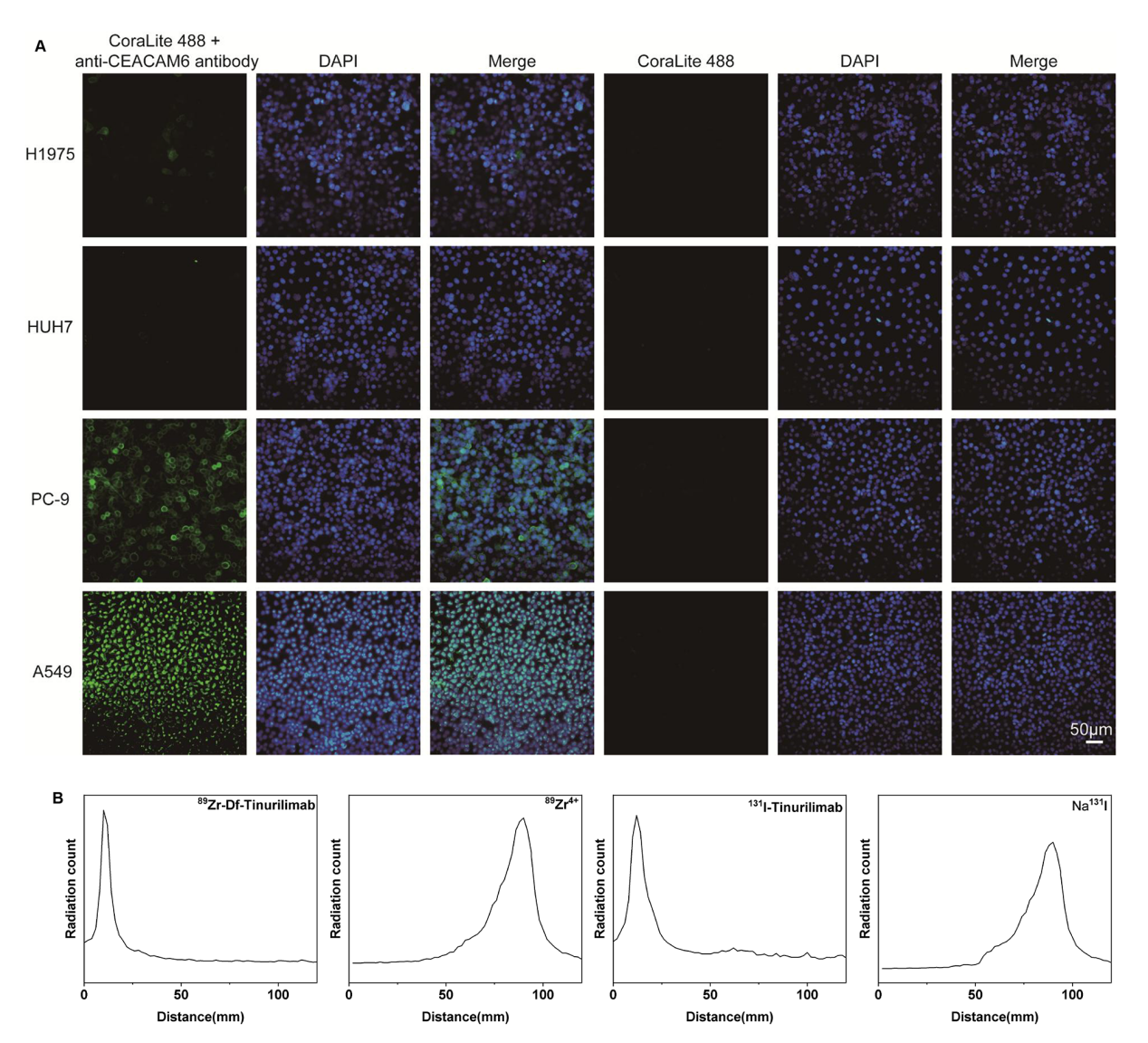
**

**Supplementary Figure 2. Immunofluorescence analysis of CEACAM6 expression and determination of the labeling yield of** **^89^Zr/^131^I labeled Tinurilimab.** (A) The Immunofluorescence analysis of CEACAM6 expression in tumor cell lines of Huh7, H1975, PC9 and A549. (B) The determination of the labeling yield of ^89^Zr/^131^I labeled Tinurilimab through radioactive high-performance liquid phase analysis.


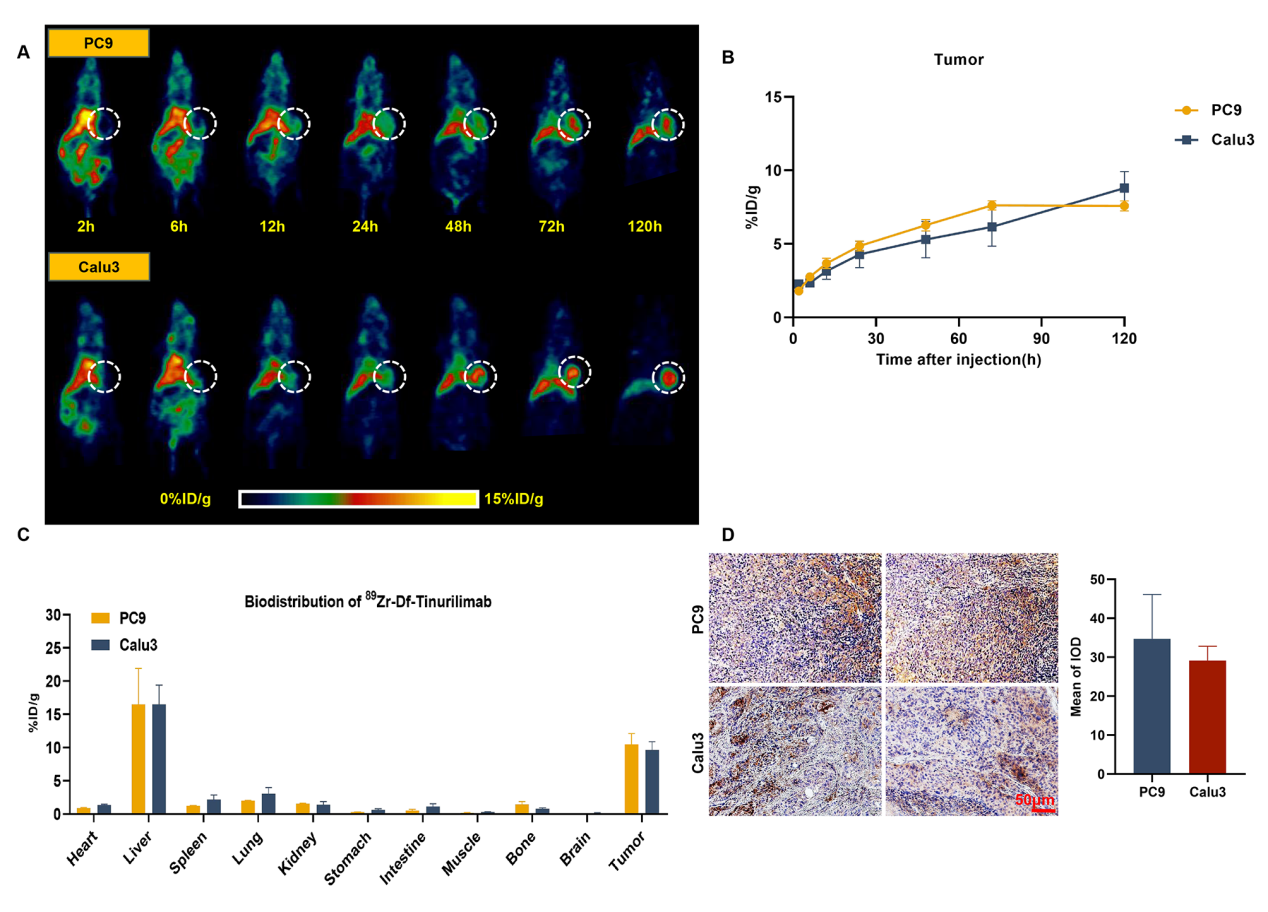


**Supplementary Figure 3. ^89^Zr-Df-Tinurilimab exhibited specific uptake in PC9 and Calu3 tumor-bearing mice.** (A) The maximum intensity projection (MIP) PET images depicting the uptake of ^89^Zr-Df-Tinurilimab in CECAMA6-positive tumor (PC9 and Calu3). (B) Quantitative analysis the ROI (region of interest) obtained from PET imaging in groups of PC9 and Calu3. (C) Ex vivo biodistributions of ^89^Zr-Df-Tinurilimab in these experiment groups at 120 h post-injection. (D) Immunohistochemical analysis of CEACAM6 expression in tumor tissues of PC9 and Calu3. All data are expressed as mean ± SEM. n = 4 for each group.


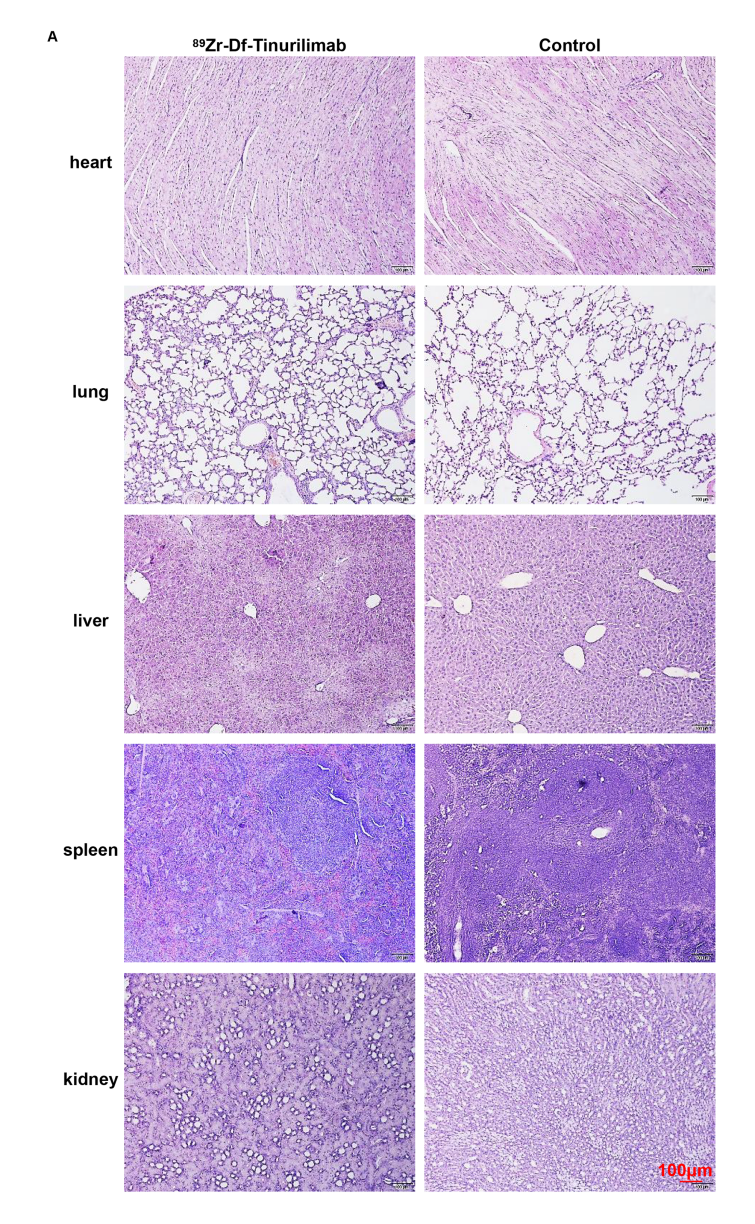


**Supplementary Figure 4. Representative images of HE staining of A549 model mice after ^89^Zr-Df-Tinurilimab injection.** n = 4 for each group.


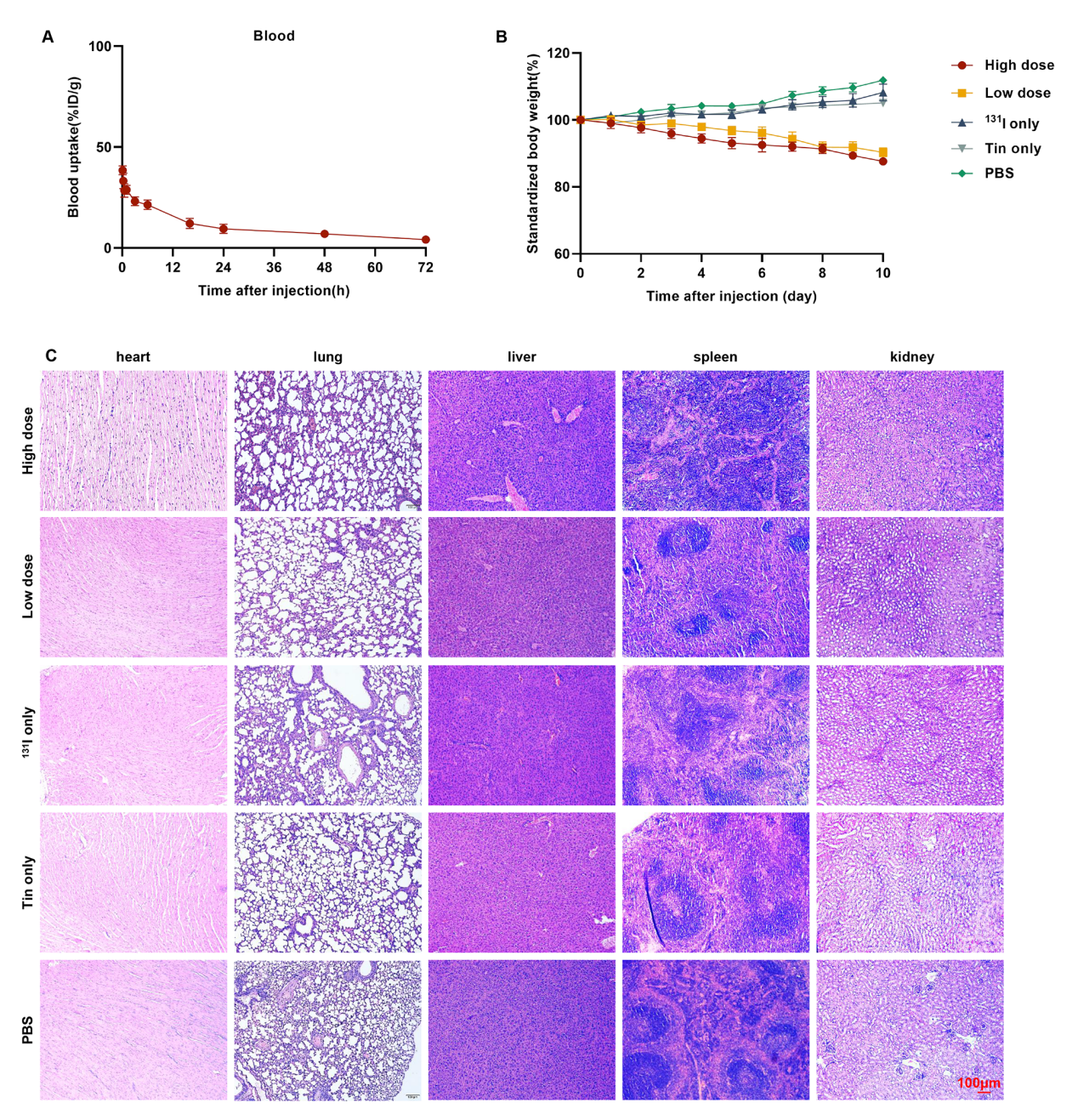


**Supplementary Figure 5. Administration of ^131^I-Tinurilimab resulted in a minor toxic effect in A549 model mice on the 10th day following initial treatment.** (A) The blood pharmacokinetics of ^131^I-Tinurilimab in A549 tumor models. (B) The standardized body weight of different treatment groups in A549 model mice. (C) Representative images of HE staining in heart, lung, liver, spleen, and kidney of ^131^I-Tinurilimab treated A549 model mice. All data are expressed as mean ± SEM. n = 8 for each group.


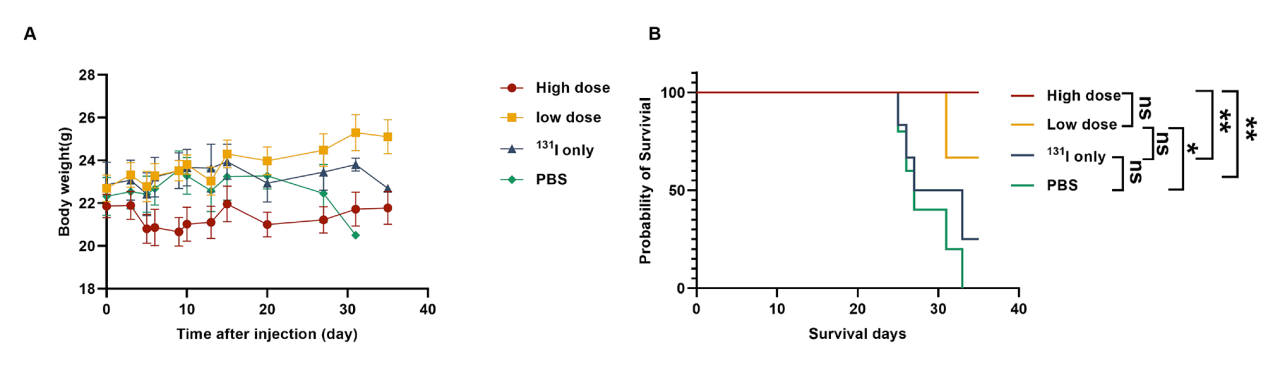


**Supplementary Figure 6. Antitumor treatment study of ^131^I-Tinurilimab in A549 tumor-bearing mice.** (A) Body weight of A549 model mice at 35th day after ^131^I-Tinurilimab treatment. (B) Survival curve of mice in the ^131^I-Tinurilimab therapy groups. All data are expressed as mean ± SEM. *p<0.05,**p < 0.01. n.s. was represented not significant. n = 5 for each group.
